# Supplementary material for: Analysis of synthetic cellular barcodes in the genome and transcriptome with BARtab and bartools
Source: Cell Rep Methods. 2024 Apr 25;4(5):100763. doi: 10.1016/j.crmeth.2024.100763 (PMC11133760; doi:10.1016/j.crmeth.2024.100763)
Supplement: Document S1. Figures S1–S6 and Table S1 [file mmc1.pdf]

Cell Reports Methods, Volume *4*

Supplemental information

Analysis of synthetic cellular barcodes in the genome and transcriptome with BARtab and bartools

Henrietta Holze, Laure Talarmain, Katie A. Fennell, Enid Y. Lam, Mark A. Dawson, and Dane Vassiliadis

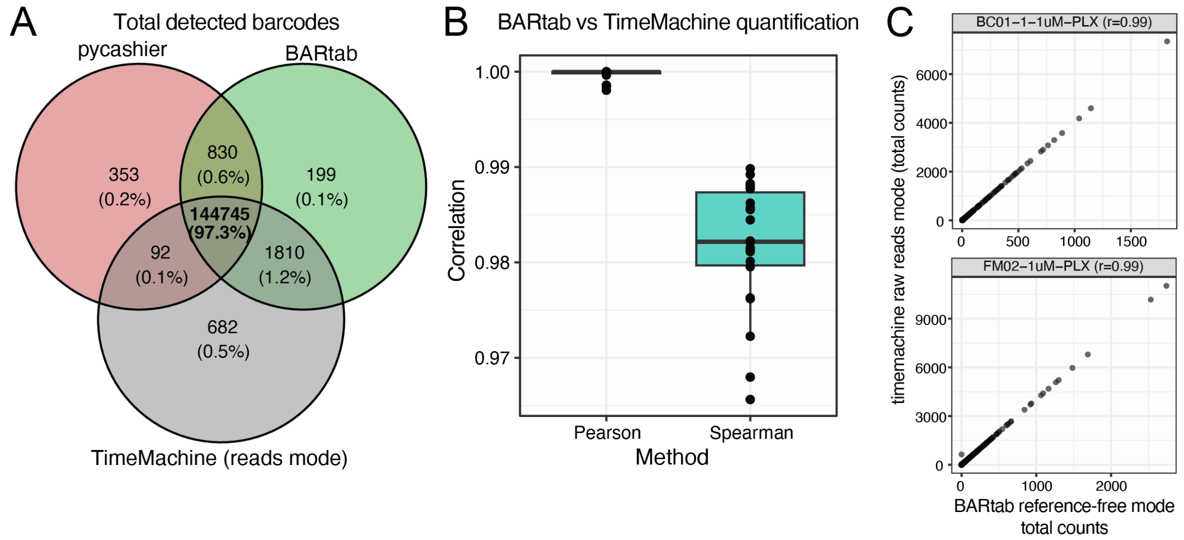

Figure S1. BARtab performance comparison – population-level data. Related to Figure 2. A) Venn diagram of total barcodes detected by BARtab, pycashier and TimeMachine (reads mode) across the 22 samples of the Goyal et al. dataset after filtering barcodes below 0.001% within a sample. **B)** Pearson and Spearman correlation of BARtab barcode quantification compared to TimeMachine (reads mode). Boxplots indicate mean and interquartile range with whiskers extending 1.5x the IQR. All samples are shown as individual points. **C)** Exemplar scatter plots for two samples from the Goyal et al. dataset showing total counts per barcode from BARtab and TimeMachine, Spearman's rank correlation coefficient in parentheses (reads mode).

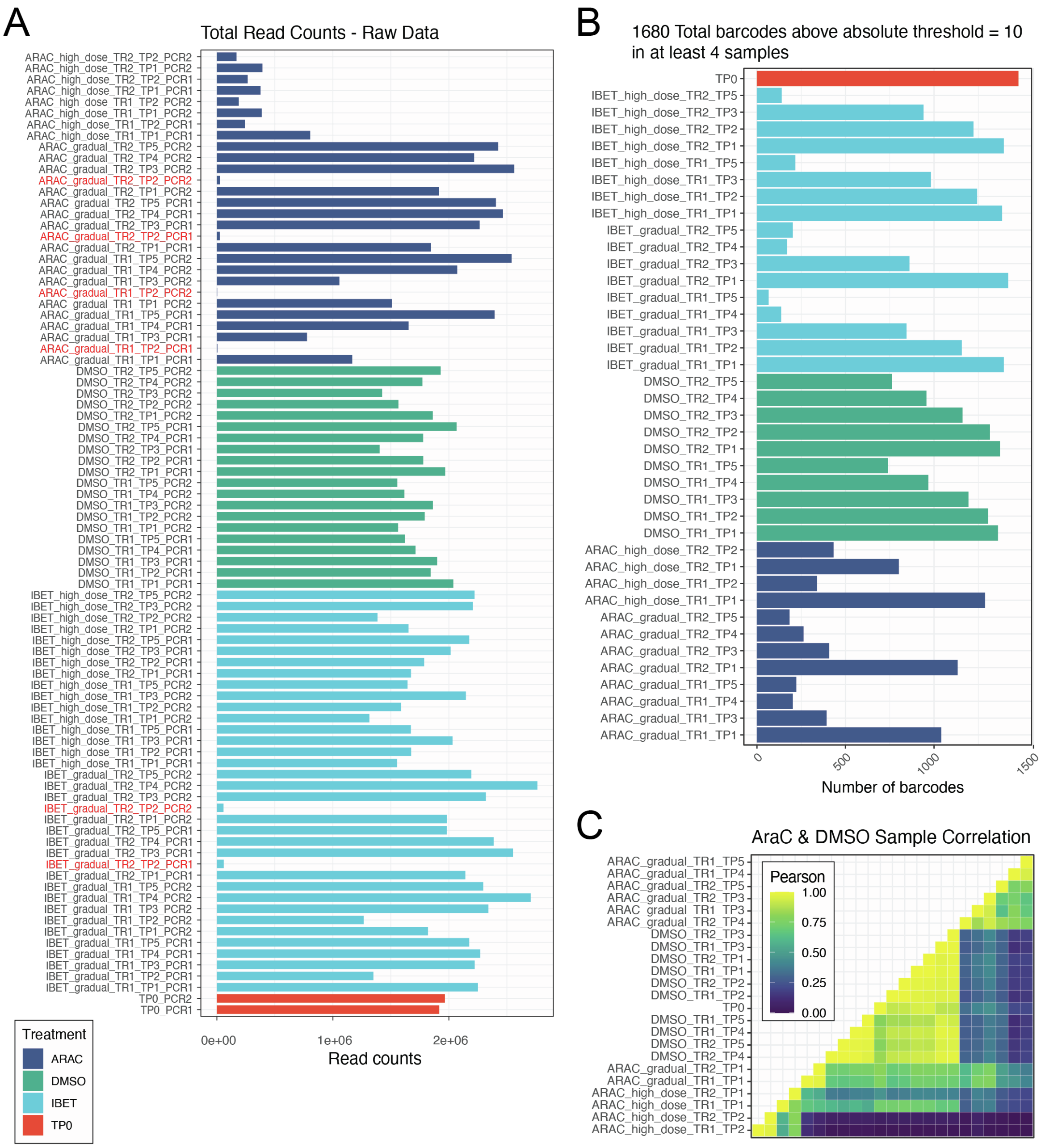

Figure S2. Additional quality control metrics for the dose escalation dataset. Related to Figure 2. A) Total number of read counts per sample for the dose escalation dataset. Poor quality samples identified using 5th percentile-based outlier detection are highlighted in red. **B)** Total number of lineage barcodes detected per sample post quality control and filtering steps for the dose escalation dataset. **C)** Pairwise Pearson correlation heatmap of biological replicate samples (indicated by TR1/2) for AraC and DMSO treatment groups in the dose escalation dataset. TR = technical replicate. TP = time point. TP0 = time point 0 / baseline sample.

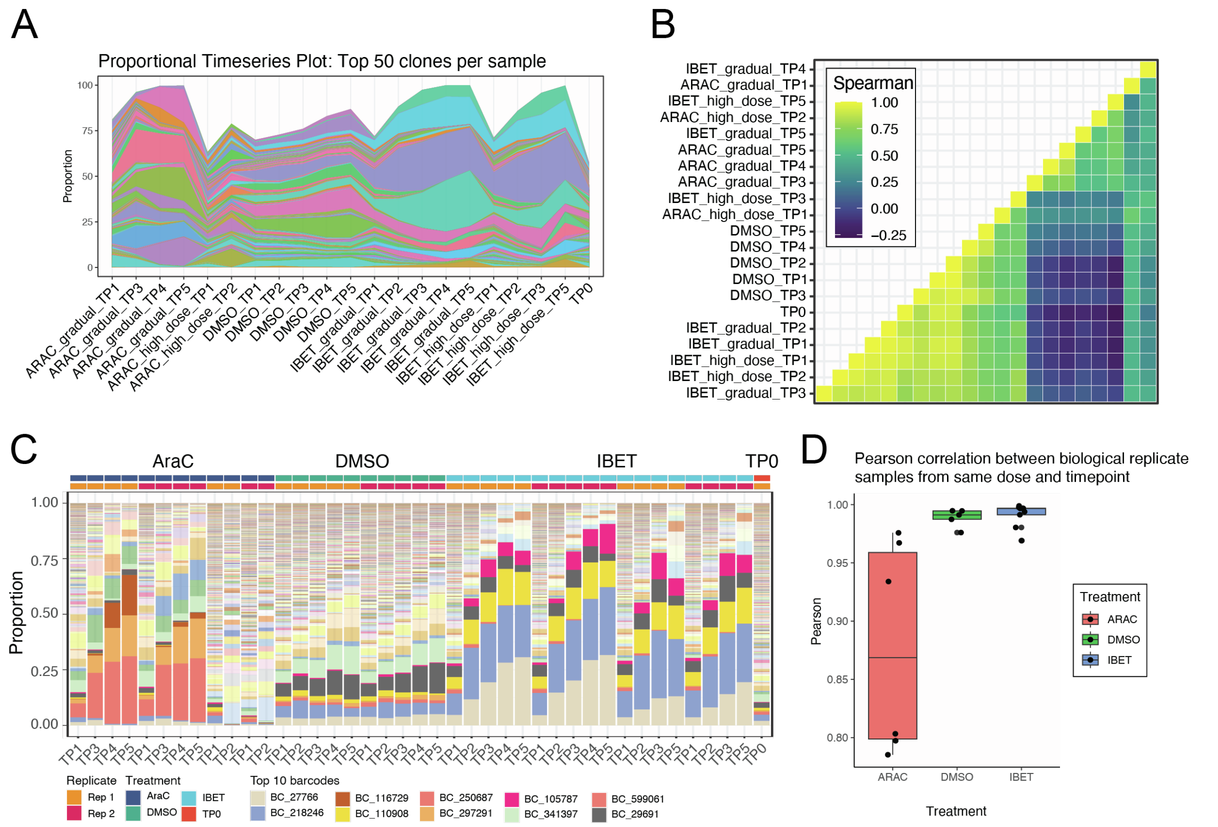

Figure S3. Additional global barcode abundance analyses for the dose escalation dataset. Related to Figure 3. A) Timeseries plot of the most abundant 50 barcodes across all samples within biological replicate 1 from the dose escalation dataset. **B)** Sample-sample pairwise Spearman correlation matrix of the dose escalation dataset, hierarchically clustered according to sample similarity. **C)** Stacked histogram of proportional barcode abundance for each sample in the dose escalation dataset. Treatment and replicate groups are indicated by the rows above the plot, the timepoint is indicated on the x-axis. The top 10 most abundant clones across the entire dataset are indicated. **D)** Boxplot of Pearson correlations between matched samples from replicate 1 and replicate 2 of the dose-escalation dataset for each of the three treatment groups. Boxplots show the mean and inter-quartile range (IQR). Whiskers extend 1.5x the IQR. Points indicate Pearson correlation values for individual pairs of samples. TP = time point. TP0 = time point 0 / baseline sample.

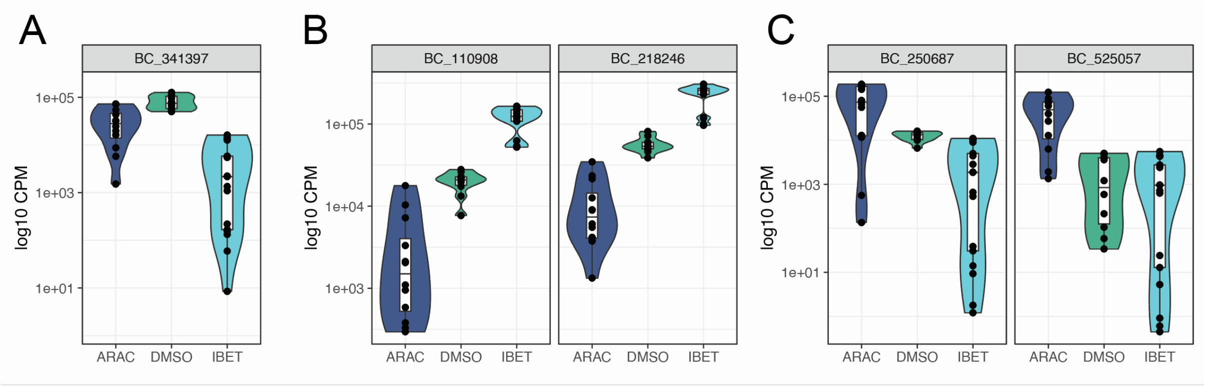

Figure S4. Individual barcode-level analysis. Related to Figure 3. Violin plots of log10 transformed counts per million (CPM) for selected barcodes from the dose escalation dataset predominant in the **(A)** vehicle (DMSO) condition, **(B)** IBET treatment condition and, **(C)** AraC treatment condition. Inset boxplots show the mean and inter-quartile range (IQR). Whiskers extend 1.5x the IQR. Points indicate CPM values for individual samples.

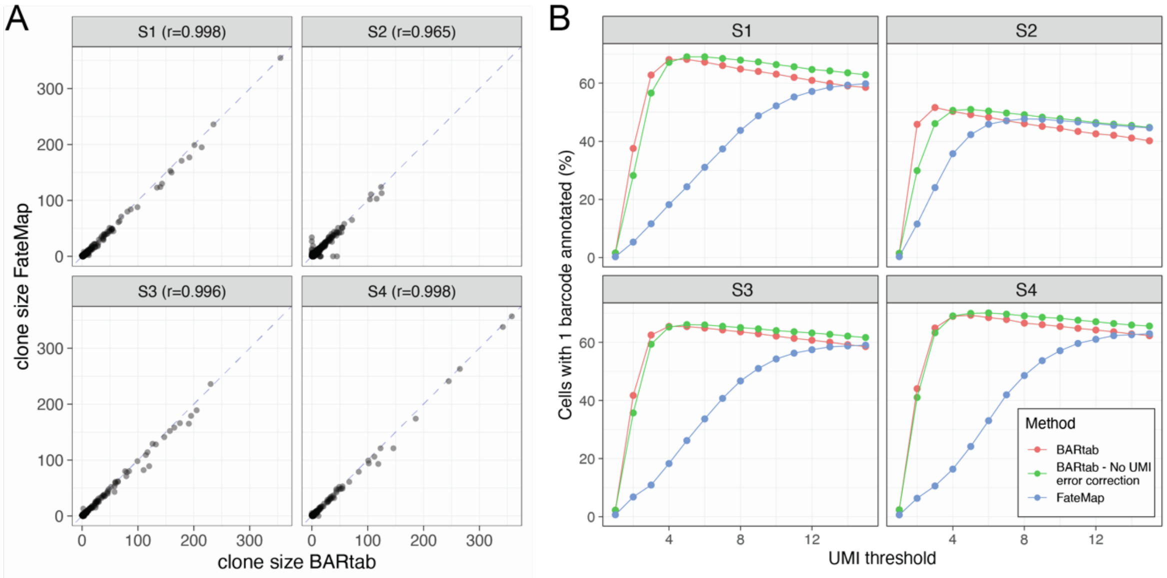

Figure S5. BARtab performance comparison – single cell data. Related to Figure 4. A) Comparison of clone sizes from single cell barcode annotation results by FateMap vs. BARtab. Samples 1-4 from the Goyal et al. 2023 FM02 dataset are shown. BARtab results are filtered for UMI threshold = 5. FateMap was run according to published parameters (UMI threshold = 15). Pearson correlation coefficient in parentheses, dashed line is x=y. **B)** Total number of cells annotated with single lineage barcodes by FateMap or BARtab across a range of UMI thresholds for each of the four samples in the Goyal et al. 2023 FM02 dataset. FateMap was run according to published parameters. BARtab was run with (red line) or without (green line) UMI sequence error correction.

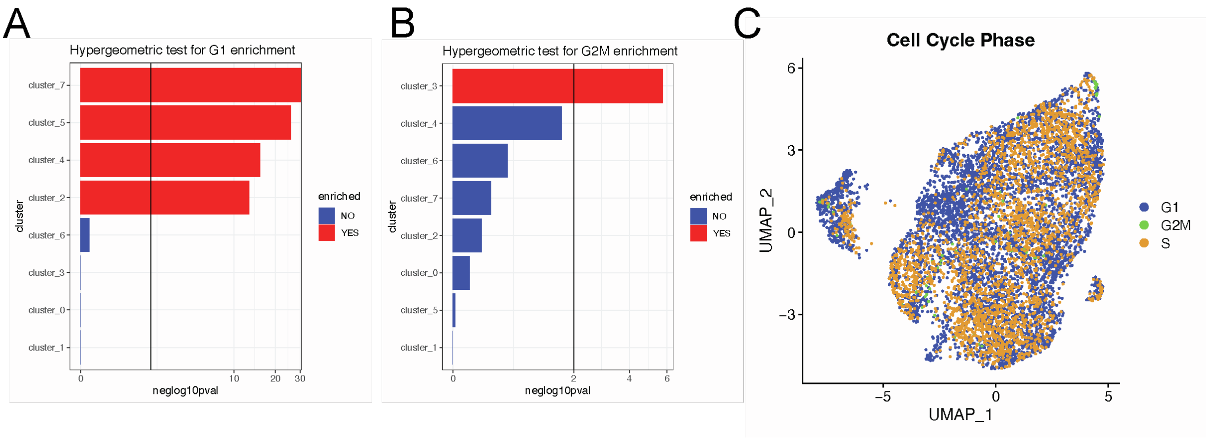

Figure S6. The plotClusterEnrichment function in *bartools* is agnostic to the grouping variable. Related to Figure 4. Hypergeometric testing for enrichment of cell cycle label G1 **(A)** and G2M **(B)** for cells within each Louvain cluster in the single-cell dataset. **C)** UMAP visualisation of the single-cell dataset with cells within each cell cycle phase highlighted.

Table S1: Example population-level cellular barcoding experiment sample sheet. Related to Figure 2.

sample	technical_rep	treatment	dose	timepoint	files
Treatment_rep1-1	1	Drug	1000nM	week_1	path/to/counts/file.txt
Treatment_rep1-2	2	Drug	1000nM	week_1	path/to/counts/file.txt
Treatment_rep2-1	1	Drug	1000nM	week_4	path/to/counts/file.txt
Treatment_rep2-2	2	Drug	1000nM	week_4	path/to/counts/file.txt
Vehicle_rep1-1	1	Vehicle	Vehicle	week_1	path/to/counts/file.txt
Vehicle_rep1-2	2	Vehicle	Vehicle	week_1	path/to/counts/file.txt
Vehicle_rep2-1	1	Vehicle	Vehicle	week_4	path/to/counts/file.txt
Vehicle_rep2-2	2	Vehicle	Vehicle	week_4	path/to/counts/file.txt
Baseline-1	1	Baseline	Baseline	Baseline	path/to/counts/file.txt
Baseline-2	2	Baseline	Baseline	Baseline	path/to/counts/file.txt
