# Supplementary material for: Analysis of synthetic cellular barcodes in the genome and transcriptome with BARtab and bartools
Source: Cell Rep Methods. 2024 Apr 25;4(5):100763. doi: 10.1016/j.crmeth.2024.100763 (PMC11133760; doi:10.1016/j.crmeth.2024.100763)
Supplement: Document S2. Article plus supplemental information [file mmc3.pdf]

Cell Reports
Methods

Article

Analysis of synthetic cellular barcodes in the genome and transcriptome with BARtab and bartools

Graphical abstract

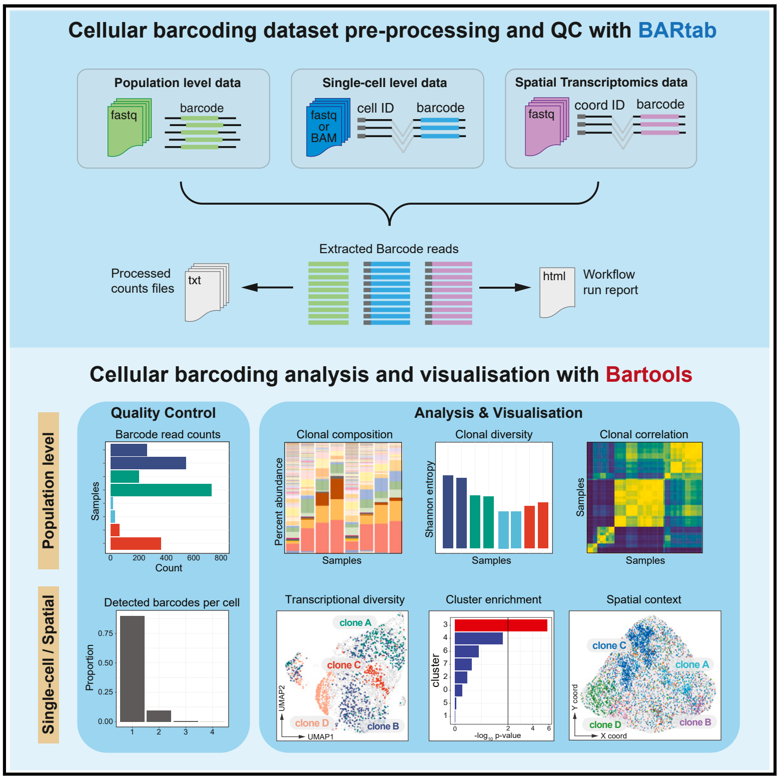

Authors

Henrietta Holze, Laure Talarmain, Katie A. Fennell, Enid Y. Lam, Mark A. Dawson, Dane Vassiliadis

Correspondence

mark.dawson@petermac.org (M.A.D.), dane.vassiliadis@petermac.org (D.V.)

In brief

Cellular barcoding methods trace the lineage relationship of individual cells at population or single-cell resolution. Holze et al. describe BARtab, a Nextflow pipeline, and bartools, an R package, that comprise an integrated software toolkit for the analysis and visualization of cellular barcoding datasets from population-level, single-cell, and spatial transcriptomics experiments.

Highlights

- BARtab and bartools comprise an end-to-end toolkit for cellular barcoding analysis
- BARtab outperforms other tools for lineage barcode extraction, QC, and enumeration
- BARtab supports population-level, single-cell, and spatial transcriptomics datasets
- bartools enables analysis and visualization of population and single-cell datasets

Check for updates

Holze et al., 2024, Cell Reports Methods *4*, 100763
May 20, 2024 © 2024 The Authors. Published by Elsevier Inc.
https://doi.org/10.1016/j.crmeth.2024.100763

CellPress

Cell Reports Methods

CellPress
OPEN ACCESS

Article

Analysis of synthetic cellular barcodes in the genome and transcriptome with BARtab and bartools

Henrietta Holze,[1,2] Laure Talarmain,[1,2] Katie A. Fennell,[1,2] Enid Y. Lam,[1,2] Mark A. Dawson,[1,2,3,*] and Dane Vassiliadis[1,2,4,*]
[1]Peter MacCallum Cancer Centre, Melbourne, VIC 3000, Australia
[2]Sir Peter MacCallum Department of Oncology, The University of Melbourne, Melbourne, VIC 3000, Australia
[3]The University of Melbourne Centre for Cancer Research, The University of Melbourne, Melbourne, VIC 3000, Australia
[4]Lead contact
*Correspondence: mark.dawson@petermac.org (M.A.D.), dane.vassiliadis@petermac.org (D.V.)
https://doi.org/10.1016/j.crmeth.2024.100763

MOTIVATION Cellular barcoding technologies are a cornerstone of the lineage-tracing field. The recent application of these methods to single-cell and spatial transcriptomics assays has necessitated the development of flexible and scalable software toolkits to analyze lineage-tracing datasets. We developed BARtab and bartools to provide a robust end-to-end solution for cellular barcoding analysis that can be readily adapted to a range of cellular barcoding approaches and experimental designs.

SUMMARY

Cellular barcoding is a lineage-tracing methodology that couples heritable synthetic barcodes to high-throughput sequencing, enabling the accurate tracing of cell lineages across a range of biological contexts. Recent studies have extended these methods by incorporating lineage information into single-cell or spatial transcriptomics readouts. Leveraging the rich biological information within these datasets requires dedicated computational tools for dataset pre-processing and analysis. Here, we present BARtab, a portable and scalable Nextflow pipeline, and bartools, an open-source R package, designed to provide an integrated end-to-end cellular barcoding analysis toolkit. BARtab and bartools contain methods to simplify the extraction, quality control, analysis, and visualization of lineage barcodes from population-level, single-cell, and spatial transcriptomics experiments. We showcase the utility of our integrated BARtab and bartools workflow via the analysis of exemplar bulk, single-cell, and spatial transcriptomics experiments containing cellular barcoding information.

INTRODUCTION

Modern lineage-tracing methods enable the accurate tracing of the progeny of individual cells across time and space. A subset of lineage-tracing techniques, termed cellular barcoding, achieve this feat by labeling individual cells with a unique genetic barcode that is heritable across cell divisions and can be subsequently read out using high-throughput sequencing technologies.[1–3] These techniques enable the investigation of clonal dynamics at unprecedented scale, helping to map developmental trajectories and lineage relationships across multiple organisms and experimental systems.[2–5] A fundamental principle of cellular barcodes is that they are heritable through cell division such that each daughter cell inherits the same barcode as its parent, thereby establishing a clonal lineage. Typically, this is achieved by engineering a unique barcode into the genome of each cell. Cellular barcoding techniques most commonly employ viral vectors or recombinant transposases to introduce a complex library of synthetic barcode sequences into the genomes of a target cell population, resulting in the unique labeling of hundreds to thousands of individual cells.[6] For population-level cellular barcoding studies, the resulting barcodes can be isolated from genomic DNA by polymerase chain reaction (PCR), sequenced using a high-throughput sequencing platform, and enumerated to reveal the frequency of each clone in a population. With the advent of single-cell sequencing technologies, recent methods have incorporated the readout of synthetic barcodes into single-cell transcriptomic datasets.[7–13] Here, synthetic barcodes are cloned into a reporter gene cassette such that they are present on mature mRNA transcripts and can be read out using poly(A)-capture-based single-cell RNA sequencing (scRNA-seq) protocols. This concept also extends to sequencing-based spatial transcriptomics

This is an open access article under the CC BY license (http://creativecommons.org/licenses/by/4.0/).

technologies that employ a similar poly(A)-based mRNA capture strategy to link gene expression with spatial context *in situ.*[14]

While cellular barcoding is a powerful methodology for understanding clonal dynamics, the analysis of barcoding datasets can be complex, causing many to turn to bespoke data analysis tools. Despite the maturity of the cellular barcoding field, there remains no accepted gold-standard data analysis pipeline or workflow suitable for population-level datasets, let alone cellular barcoding analysis from single-cell or spatial datasets.[15] Recent efforts to standardize the analysis of such data have focused primarily on dataset visualization and lack support for the upstream pre-processing of raw datasets or for the next wave of lineage-tracing studies that will utilize expressed synthetic barcodes.[16–18] Indeed, recent single-cell expressed barcoding studies incorporate their own customized analysis pipelines in a manner that lacks versatility for studies that utilize a conceptually similar yet slightly different biological workflow.[7,8,10,11,19] Thus, there is a need for an end-to-end integrated solution for cellular barcoding dataset pre-processing, analysis, and visualization that is flexible to different barcode designs and is portable and scalable across computational environments.

To support the standardization of barcode dataset pre-processing and quality control (QC), here, we introduce BARtab, a portable and scalable Nextflow pipeline that allows the generation of barcode counts tables from population-level barcode sequencing workflows, as well as barcode extraction, enumeration, and cell annotation from single-cell and spatial transcriptomics datasets. Moreover, to facilitate the downstream analysis and visualization of cellular barcoding workflows at bulk and single-cell resolution, we developed bartools, a flexible open-source R package that incorporates workflows for population-level cellular barcoding data analysis, as well as methods for single-cell expressed barcode analysis and visualization. bartools provides a convenient interface between cutting-edge methods for the analysis of cellular barcoding datasets and the robust analytical framework established within the R ecosystem. We demonstrate the improved performance and versatility of BARtab compared to other barcode pre-processing software and showcase the capabilities of our integrated BARtab and bartools workflow through the analysis and visualization of exemplar population-level, single-cell-level, and spatial-transcriptomics-based cellular barcoding datasets, which we make publicly available. Together, BARtab and bartools comprise an end-to-end integrated toolkit that will help streamline and standardize cellular barcoding experiments for the lineage-tracing field at large.

RESULTS

BARtab and bartools comprise an integrated cellular barcoding analysis workflow

Population- and single-cell-level cellular barcoding workflows are usually read out using high-throughput sequencing, resulting in raw sequence data containing barcode information that must undergo QC, barcode extraction, and quantification. This task is usually performed by bespoke software specific to the study in question. Publicly available tools that exist, such as genBaRcode,[16] pycashier,[20,21] or xcalibr,[22] are limited in their flexibility for different barcode designs and lengths, support for paired-end datasets, portability and resource allocation, support for reference-based barcode quantification, or support for single-cell and spatial transcriptomics expressed cellular barcoding datasets (Table 1). In contrast, BARtab is an open-source Nextflow pipeline[23] written as a versatile, portable, reproducible, and scalable solution for high-throughput barcode dataset pre-processing from population-level, single-cell, and spatial transcriptomics cellular barcoding experiments. BARtab leverages widely used bioinformatics tools including fastp, FLASh, cutadapt, samtools, bowtie, starcode, umi-tools, and FastQC.[24–29] In its simplest form, running on population-level cellular barcoding data, BARtab performs the following steps: (1) import and QC of raw sequence data, (2) barcode QC and filtering, (3) adapter trimming and extraction of barcodes from raw sequencing reads, (4) barcode quantification, and (5) reporting (Figure 1).

Alternative subworkflows are also available for situations where no reference library of barcodes is available, for paired-end reads that require merging prior to extraction (e.g., for barcode constructs where the length of the barcode is greater than the length of the sequenced read), or for extraction of barcode reads from single-cell and spatial transcriptomics datasets utilizing expressed barcode technology (Figure 1). For population-level datasets, the primary output of BARtab is a table of raw counts per barcode where rows are individual barcodes and columns are individual samples. For single-cell datasets, BARtab outputs a single table per sample containing unique molecular identifier (UMI) and lineage barcode information per cell ID. This table can be imported as sample metadata into established R- or Python-based scRNA-seq analysis packages such as SingleCellExperiment from the Bioconductor project, Seurat, or Scanpy.[31–33] For bulk-level data, the counts table output from BARtab is pre-formatted for, and can be easily read into, bartools, thus connecting dataset pre-processing to downstream analysis and visualization.

bartools is an open-source R package that accepts tables of raw counts (with individual barcodes/tags as rows and samples as columns), such as those generated by BARtab, or other software as input. By utilizing the edgeR DGEList framework,[34] barcode count datasets can be read into bartools as individual counts table files or as defined in an experimental sample sheet, which organizes raw count data alongside associated sample metadata (see Table S1 for an example). QC metrics including the results of filtering and reference mapping stages from an associated BARtab pre-processing run can be plotted per sample in bartools using the plotBARtabFilterQC() and plotBARtabMapQC() functions. Following initial dataset filtering and QC, cellular barcoding datasets can be further processed using built-in functions that perform sample normalization, downstream abundance and diversity analysis, and visualization of synthetic barcode data (Figure 1). The bartools package also includes analysis methods that accept single-cell objects containing cellular barcoding information in Seurat or SingleCellExperiment format, which can be used to aid in QC and assess clone-level properties in concert with popular scRNA-seq analysis workflows (Table 2). Overall, the integrated combination of BARtab and bartools improves upon other

Table 1. Feature comparison of BARtab and other barcode pre-processing tools

	BARtab	pycashier[21]	Rewind/TimeMachine/FateMap[13,30]	genBaRcode[16]	xcalibr[22]
Link	https://github.com/DaneVass/BARtab	https://github.com/brocklab/pycashier	https://github.com/arjunrajlaboratory/TimeMachine	https://cran.r-project.org/web/packages/genBaRcode/index.html	https://github.com/NKI-GCF/xcalibr
Implementation	Nextflow (Groovy)	Python	Python	R	Perl
Input type	Fastq, BAM	Fastq	Fastq	Fastq	Fastq
Dataset support	population, single cell, spatial	population, single cell	population, single cell	population	population
Flexibility for barcode design	yes	yes	no (hard-coded adapter)	yes	yes
Barcode length flexibility	yes	yes (limited)[a]	yes (limited)[b]	yes	no
Supports paired-end datasets	yes	yes	no	no	no
Filtering method	minimum Phred score in percentage of read	minimum Phred score in percentage of read	<5 positions upstream of barcode below Phred score	average Phred score	N/A
Portability	Singularity, Docker, Conda	Conda, Docker	N/A	CRAN	N/A
Process-specific resource allocation	automated (via Nextflow)	manual	manual	manual	manual
Resource allocation	automated (via Nextflow)	manual	manual	manual	manual
Reference-based quantification	yes	no	no	no	no
Reference-free clustering	yes	yes	yes	yes	yes
Sample parallelization	yes (via Nextflow)	no	no	no	no
QC report	read quality, adapter trimming, and alignment	read quality	N/A	N/A	N/A

N/A, not applicable.
[a]Barcode length flexibility is limited to defined barcode length ± allowed Levenshtein distance for barcode clustering.
[b]Barcode length flexibility is limited by the detection of barcode flanking regions in specific regions of the read.

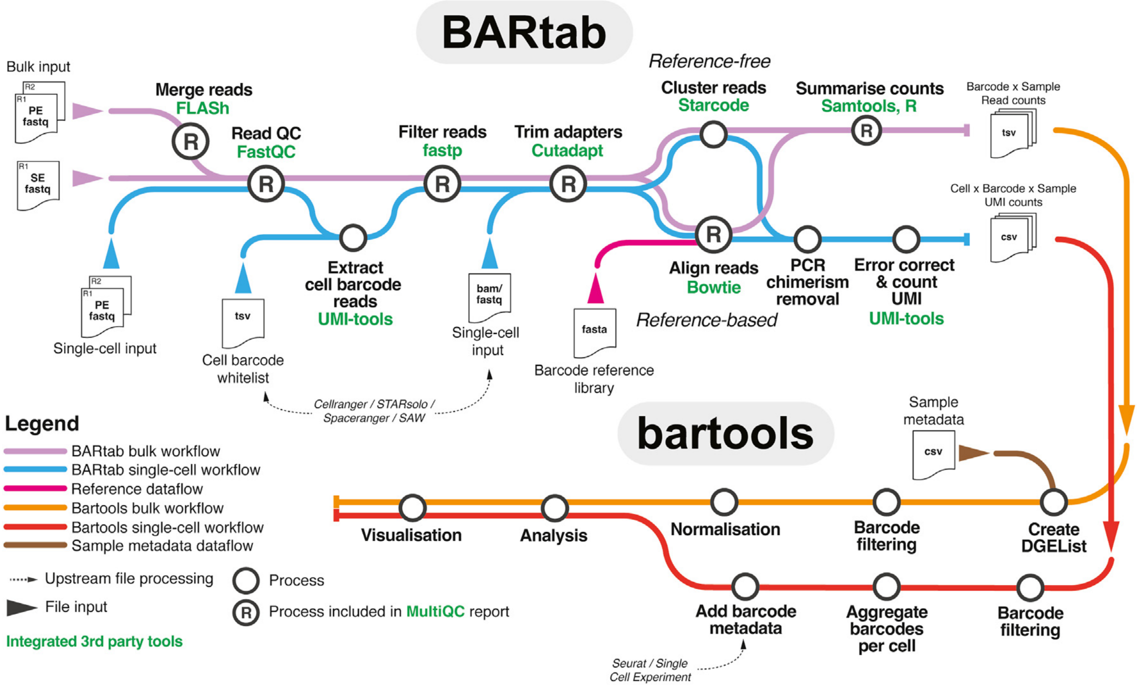

Figure 1. The BARtab and bartools workflow for high-throughput cellular barcoding analysis

BARtab is a Nextflow pipeline to process high-throughput sequencing datasets containing cellular barcode information. BARtab contains two main subworkflows that permit the analysis of (1) population-level cellular barcoding data (“bulk workflow”) and (2) single-cell datasets (“single-cell workflow”). For the bulk workflow, BARtab takes single or paired-end datasets in fastq format as input and performs read merging (paired-end only), quality filtering, and adapter trimming. Filtering and error correction of barcodes can occur using a reference-based (recommended) strategy, which aligns putative barcode sequences to a user-supplied reference of known lineage barcodes, or by a reference-free workflow, which clusters and merges barcode sequences within a defined Levenshtein distance. Barcodes are then quantified per sample by read counts. For the single-cell workflow, raw paired-end datasets in fastq format (e.g., from targeted amplification of cellular barcodes within a single-cell library) or reads in BAM format after alignment to a reference genome (output as part of the pre-processing of scRNA-seq data) are taken as input. Barcode-containing reads are selected, quality filtered, and finally quantified by UMI counts using either a reference-based or reference-free approach. Outputs of all workflows include tables of processed counts with individual barcodes as rows and samples as columns, as well as a MultiQC run report including read filtering and alignment metrics. Tables of counts from BARtab, alongside associated sample metadata, can be imported into bartools for further downstream analysis and visualization. For single-cell datasets, barcode import interfaces directly with popular R-based single-cell frameworks including Seurat and SingleCellExperiment, resulting in cell-level annotation with lineage barcode metadata, which can be leveraged for analysis and visualization in bartools and other single-cell analysis packages.

available dataset pre-processing and analysis/visualization software by providing an end-to-end analysis solution that improves upon previous offerings and permits analysis of single-cell and spatially expressed cellular barcoding datasets.

BARtab improves upon previous cellular barcode dataset pre-processing software

Cellular barcoding datasets are prone to technical artifacts that skew the representation and perceived abundance of barcoded clones of interest. Barcode amplification by PCR, high-throughput sequencing, read filtering, and downstream analysis stages all present potential sources of technical error in a cellular barcoding workflow.[35,36] In BARtab, we encourage a reference-based approach for the quantification of cellular barcoding datasets by mapping putative barcode containing reads to a known reference set of accepted barcodes.[1,2,9] This reference set of barcodes can be obtained via deep sequencing of the library plasmid pool, enabling comprehensive identification of true barcodes present within a particular library without overt reliance on sequence error correction processes. In contrast, similar approaches including genBaRcode,[16] pycashier,[20,21] or xcalibr (https://github.com/NKI-GCF/xcalibr) take a distance threshold approach to combine similar barcodes together that can arise through errors during PCR or sequencing.[16,17] Although these approaches sidestep the requirement of a deeply sequenced reference, further QC and filtering procedures are recommended to eliminate spurious results. Alongside the reference-based workflow, BARtab can also perform reference-free identification and quantification of barcodes from single-cell and bulk datasets by employing a Levenshtein-distance-based clustering approach implemented in Starcode.[24] In summary, BARtab allows for reference-based as well as reference-free extraction of barcodes from both bulk and single-cell datasets, is flexible toward barcode design and sequencing quality, provides a detailed quality report, and is portable and parallelized to facilitate large-scale data processing.

To demonstrate the versatility of BARtab, we compared its performance to pycashier and Rewind/TimeMachine, two

CellPress
OPEN ACCESS

Table 2. Feature comparison of bartools and other barcode analysis toolkits

	bartools	genBaRcode[16]	barcodetrackR[17]	CellDestiny[18]
Data structure	DGEList (edgeR)	custom R object	SummarisedExperiment	Rshiny
Normalization modes	percentage of abundance, CPM, TMM	N/A	percentage of abundance, CPM	percentage of abundance
QC and aggregation of replicates	yes	no	no	yes
Clone size analysis	yes	no	yes	yes
Support for single-cell experiments	yes	no	no	yes
Rshiny app	no	yes	yes	yes

TMM, trimmed mean of M values.

recently published toolkits for cellular barcode extraction.[13,21,30] Using each tool, we re-analyzed an exemplar population-level cellular barcoding dataset consisting of 22 individual samples from a recent Rewind/TimeMachine study by Goyal et al.[30] Since the pycashier and Rewind/TimeMachine approaches do not support reference-based barcode quantification (Table 1), we ran BARtab using the reference-free clustering approach. We used default parameters for the Rewind/TimeMachine tool as per the original publication and parameters for pycashier and BARtab that matched those analysis conditions as closely as possible to allow comparable barcode quantification performance.

We observed a total of 144,745 barcodes (97.3%) with at least 0.001% frequency within a sample detected by all three tools across the 22 samples. The overlap of barcode identification between BARtab and TimeMachine was slightly improved compared to pycashier and TimeMachine (1,810 barcodes [1.2%] were only detected by TimeMachine and BARtab, 92 barcodes [0.1%] were only detected by TimeMachine and pycashier [Figure S1A]). 1,807/1,810 (99.8%) of barcodes detected by BARtab and TimeMachine alone were outside the allowed barcode length range of pycashier when run using comparable settings, suggesting that the improved overlap of barcodes detected by BARtab with those detected by TimeMachine is due to greater flexibility in accepted barcode lengths compared to pycashier (see STAR Methods). We also examined barcode quantification concordance of BARtab to the published dataset. Here, we observed striking concordance in barcode representation and abundance with Pearson and Spearman correlation values between the two tools greater than 0.96 for all samples (Figures S1B and S1C). Finally, we compared the runtimes of BARtab, pycashier, and Rewind/TimeMachine across the 22 sample dataset on a computing cluster with 32 GB memory and 20 CPUs allocated to each workflow. All tools consumed less memory than was allocated: BARtab (6.83 GB), TimeMachine (3.52 GB), and pycashier (1.81 GB). BARtab and pycashier performed comparably with respect to runtime, processing all datasets in 35 and 36 min, respectively, while Rewind/TimeMachine took 4 h 59 min. The difference in runtime can be attributed to the lack of parallelization and resource allocation support in TimeMachine causing all samples to be run serially. Together, these analyses highlight the versatility of BARtab for different cellular barcoding methodologies and its improved sensitivity and processing speed compared to currently available software.

bartools supports the QC of population-level cellular barcoding datasets

Next, to further demonstrate the capabilities of BARtab and bartools, we generated an exemplar population-level cellular barcoding dataset using the single-cell profiling and lineage tracing (SPLINTR) lineage-tracing system.[9,37] Here, acute myeloid leukemia (AML) cells were cultured in the presence of gradually escalating doses or an upfront high dose of cytarabine (AraC), a conventional chemotherapy used routinely in the clinic, or IBET-151 (IBET), a targeted epigenetic therapy against the BET (bromodomain and extra-terminal domain-containing) family of transcriptional co-activators that has shown pre-clinical efficacy against several AML subtypes[37] (Figure 2A). This "dose-escalation" dataset comprises population-level barcode-seq data per dose and time point.

Following the pre-processing of raw reads into barcode counts and their import into bartools, samples can be filtered according to absolute and relative (proportion-based) thresholds. Total read depth and number of detected barcodes can then be assessed per sample. To demonstrate this, we applied both sample-level and barcode-level filters to the dose-escalation dataset. First, we filtered samples using a 5th percentile outlier threshold calculated from the total read counts across samples. This eliminated six low-quality samples that were unlikely to yield reliable data (Figure S2A). Next, using the thresholdCounts() function, we assessed different relative and absolute read count thresholds on a per-barcode basis and chose to apply a low-stringency filter, removing barcodes from the dataset with less than 10 reads across at least four samples. This eliminated 110 low-abundance barcodes from the dataset that are likely the result of sequencing errors or other technical noise. This filtering resulted in a dataset of 1,680 high-confidence barcodes across 41 samples. In line with our earlier findings in this model system, we observed a gradual decrease in total barcode numbers across the vehicle treatment time course, suggesting that gradual clonal drift results in the outgrowth of more proliferative clones over time under steady-state conditions. In contrast, total barcode numbers declined more rapidly across the IBET-151 and AraC treatment time courses, indicating clonal restriction due primarily to the selective pressure of therapy (Figure S2B).[37]

If the experimental design incorporates technical replicates, which we also encourage, further QC can be performed using calcReplicateCorr() to assess replicate correlation and removing samples with correlation scores below a reasonable threshold (Figure 2B). Poor technical replicate correlation scores can

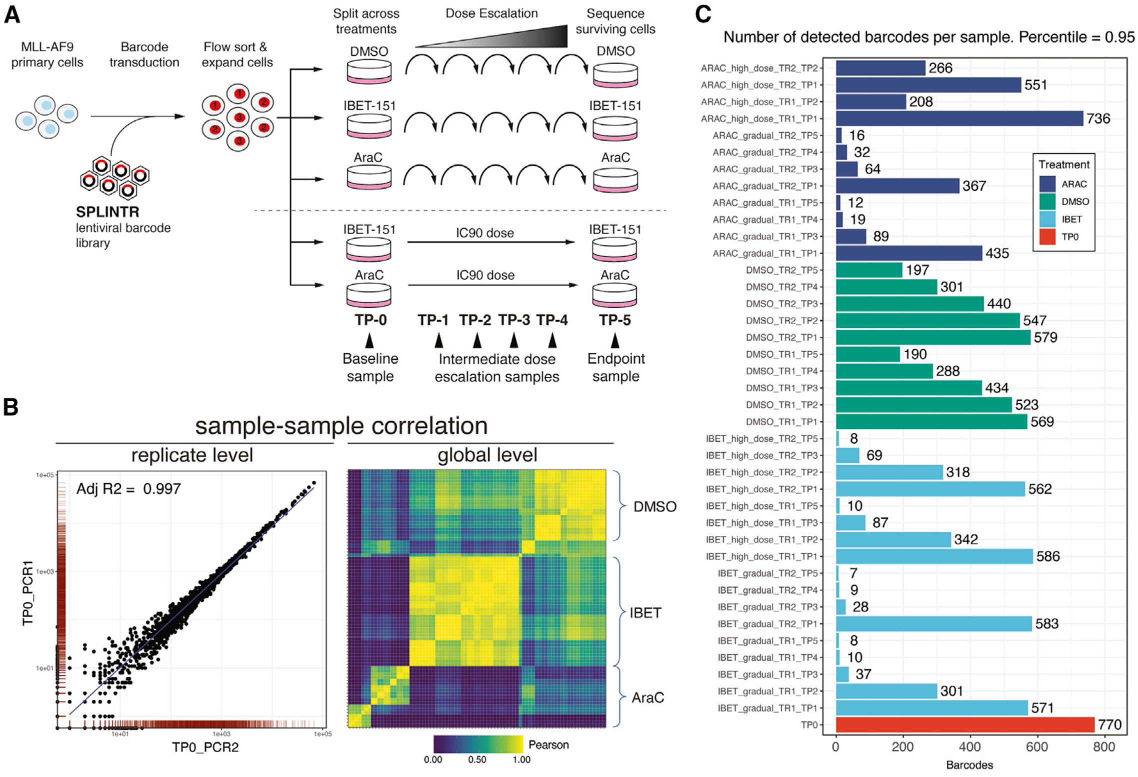

Figure 2. Cellular barcoding QC analysis of the dose response dataset

(A) Schematic of dose-escalation dataset experimental design. Drug-naive MLL-AF9 cells were transduced with a SPLINTR barcode-encoding lentiviral library in liquid culture, flow sorted for barcode expression, and expanded for 1 week in liquid culture. Following expansion, barcoded clones were split evenly into media containing either 400 nM IBET-151, 300 nM cytarabine (AraC), or 0.1% (v/v) DMSO (vehicle control) such that each treatment arm received an identical barcode repertoire. Cells were re-plated weekly in escalating concentrations of IBET-151, AraC, or vehicle. Alongside the dose escalation, the same pool of barcoded cells were seeded into an IC90 dose of IBET-151 (800 nM) or AraC (700 nM). Barcode sequencing was performed on samples obtained at the time points indicated. The experiment was performed in biological duplicate. For the AraC high-dose group, only time points 1 and 2 were sequenced due to low cell viability at later time points.

(B) Replicate and global-level sample correlation analyses. Left: representative scatterplot of barcodes in technical replicates for the TP0 sample. Right: pairwise Pearson correlation heatmap of all biological replicate samples (treatment group indicated) in the dose-escalation dataset.

(C) Total number of barcodes present at the 95th percentile in each sample following sample QC and merging of technical replicates. TR, technical replicate; TP, time point; TP0, time point 0/baseline sample.

indicate sampling bias during sample preparation or sequencing coverage issues that may complicate dataset interpretation. Furthermore, when examined on a global level, correlation scores between samples and replicates can reveal high-level insights into clonal structure. For example, in the dose-escalation dataset, pairwise correlation analysis performed using plotBarcodeCorrelation() showed that AraC high-dose (IC90) and gradual dose-escalation treatment samples clustered independently, indicating the selection of distinct groups of clones by the two treatment strategies (Figure S2C).

Following sample QC and filtering, technical replicates displaying greater than 90% correlation at the sample level were averaged using collapseReplicates(), and the number of barcodes comprising the 95th percentile in each remaining sample was calculated using the calcPercentileBarcodes() and plotDetectedBarcodes() functions (Figure 2C). Finally, dataset normalization was performed using a counts per million transformation via normaliseCounts(). Trimmed mean of M-values-[38] and percentage-abundance-based metrics are also available as normalization options within bartools. When applied correctly, these QC and normalization approaches result in a clean cellular barcoding dataset that is ready for downstream analysis and visualization.

bartools enables visualization and comparison of global and individual clone abundance

Following dataset normalization, global visualization of cellular barcoding data is useful to understand the overall clonal composition of within and between samples. bartools contains several visualization options to assess global clonal repertoires. For example, bubble plots (via plotBarcodeBubble() and plotOrderedBubble(); Figure 3A) give an overview of relative sample composition while informing on the proportional abundance of clones and the identities of overrepresented clones within samples. Heatmaps (via plotBarcodeHeatmap()), similar to those implemented in the barcodetrackR package,[17] allow similar global overview and stratification of samples based on multiple metadata fields but are simpler to interpret on a subset of the total

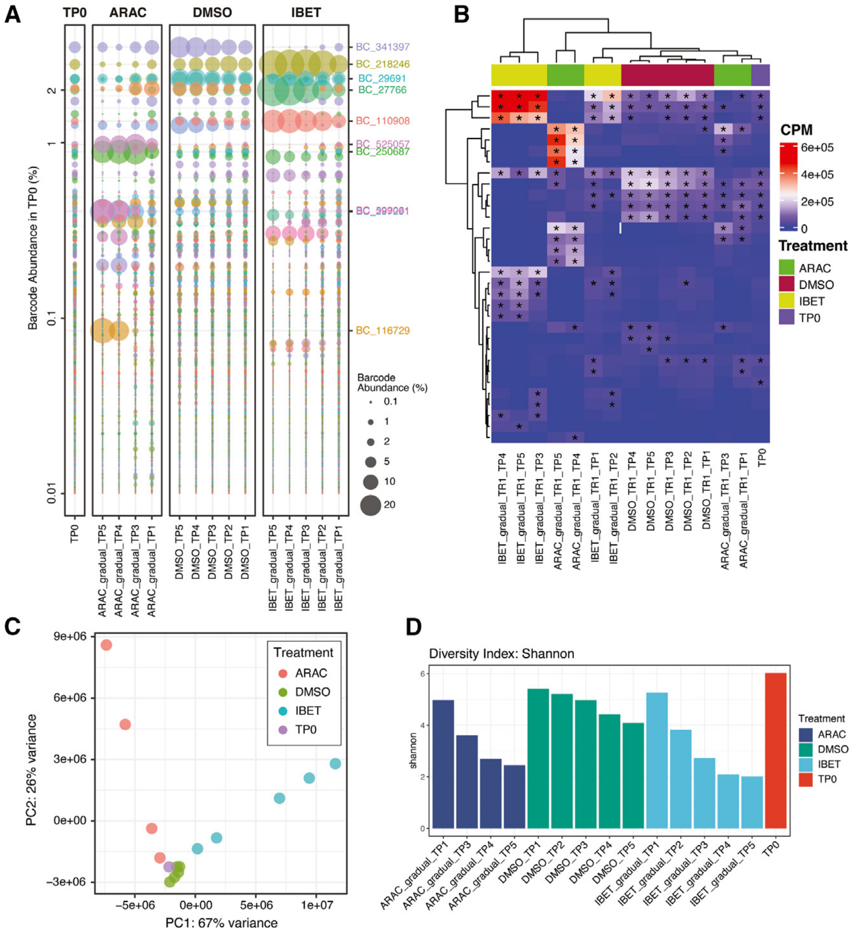

Figure 3. Analysis of global clonal repertoires, sample correlation, and diversity in the dose-escalation dataset

(A) Bubble plot of a subset of samples from replicate 1 of the dose-escalation dataset ordered in descending rank order according to percentage of abundance in the baseline (TP0) sample. Lefthand side y axis indicates percentage of abundance of each clone in the reference sample (TP0). Right-hand y axis highlights clones present at a percentage of abundance above 5% in at least one sample.

(B) Heatmap of a subset of replicate 1 samples from the dose-escalation dataset showing counts per million (CPM) values for the top 10 most abundant barcodes (rows) in each sample (columns). Starred cells indicate barcodes that are among the top 10 most abundant within that sample.

(C) Principal-component analysis (PCA) of the dose-escalation dataset. Vehicle and drug (IBET-151 or AraC) treatment conditions are clearly separated across PC1.

(D) Histogram of Shannon diversity for baseline TP0 and vehicle- (DMSO) and drug-treated (IBET-151 or AraC) samples at each time point within biological replicate 1 from the dose-escalation dataset. TR, technical replicate; TP, time point; TP0, time point 0/baseline sample.

dataset (Figure 3B). Time course studies such as the dose-escalation dataset can additionally benefit from timeseries plots, which can reveal the temporal nature of clonal fitness (plotBarcodeTimeseries(); Figure S3A). Further to analyses of clone abundance, it is also of interest to calculate correlation or relatedness scores between samples in a barcoding dataset. bartools leverages gold-standard linear methods to determine sample similarity including principal-component analysis (PCA), sample-sample distance, and correlation methods (Figures 3C and S3B). Moreover, bartools incorporates functions from the R packages vegan and ineq[39,40] to calculate various population diversity metrics including the Shannon, Simpson, Inverse Simpson, and Gini indices (Figure 3D).

To gain further insight into the clonal dynamics of drug resistance in the dose-escalation dataset, we performed global analyses of the quality controlled and normalized dose-escalation dataset using bartools. These analyses revealed clear outgrowth of distinct groups of clones in the vehicle- and either drug-treated conditions (Figures 3A and 3B). By PCA, treatments were clearly separated along PC1, with the dose-escalation time course separated along PC2 (Figure 3C). These differences were also observed in correlation and diversity analyses across samples, with later time points in the drug and vehicle conditions both less diverse and less well correlated with the baseline and early dose-escalation samples (Figure 3D).

We next considered the reproducibility in clonal profiles between biological replicates. Using the plotBarcodeHistogram() function to provide a qualitative overview of the dataset, we found that replicate samples displayed similar overall clonal composition across all treatments and time points (Figure S3C). We next calculated Pearson correlation values for pairs of treatment samples across both biological replicates for each time point. This revealed a strong correlation for both DMSO and IBET-151 treatments (all > 0.95) with a slight reduction for AraC-treated samples, which could reflect the intensive cytotoxic effects of this therapy (Figure S3D). These analyses reveal that IBET-151 or AraC therapy reproducibly selects for distinct groups of clones, demonstrating the utility of bartools to extract biological insight from cellular barcoding datasets.

Following global-level analyses of clonal composition within samples, it is of interest to interrogate the relative fitness of individual barcodes across samples or treatment groups. Such clone-level visualizations are also supported in bartools and can be useful to specifically compare clones of interest relative to others across samples or conditions. Using the plotBarcodeBoxplot() function, normalized abundance of individual clones can be displayed across sample metadata conditions. For the dose-escalation dataset, these analyses revealed striking differences in clonal fitness across the vehicle and treatment conditions, with two classes of clones emerging: those that display increased fitness in vehicle conditions (Figure S4A) and those that display increased fitness in either drug treatment (Figures S4B and S4C). Overall, the combination of bulk- and

clone-level analyses afforded in bartools will enable researchers to thoroughly interrogate clonal dynamics in diverse systems and under various perturbation conditions.

BARtab and bartools facilitate single-cell expressed cellular barcoding analysis

Recently developed expressed cellular barcoding tools, such as SPLINTR,[9] Rewind/FateMap,[13,30] Clonmapper,[21] TREX,[14] Watermelon,[10] LARRY,[8] and CellTag,[7,41] utilize the 3′ capture of polyadenylated messenger RNAs in scRNA-seq libraries to capture transcripts encoding the cellular lineage barcode of each cell. Incorporation of these transcripts links cellular barcodes to a cell ID and UMI, thus labeling single-cell transcriptomes with their corresponding clonal identity. We developed BARtab to be compatible with these expressed cellular barcoding workflows. The single-cell subworkflow of BARtab accepts two types of inputs: (1) sequence data in binary alignment map (BAM) format arising from single-cell data pre-processed using the widely utilized Cell Ranger pipeline from 10× Genomics[42] or the open-source STARsolo[43] or (2) paired-end data in fastq format following targeted amplification of barcode-containing reads from single-cell library cDNA (Figure 1).

Running in single-cell mode, BARtab first extracts reads containing lineage barcode information according to user-defined parameters for barcode identification. For BAM file inputs, the cell ID and UMI are contained within the read tag information, allowing lineage barcode, cell ID, and UMI information to be extracted simultaneously. For paired-end fastq inputs arising from targeted amplicon sequencing of the single-cell library, cell ID and UMI information are extracted separately to the lineage barcode information and merged. Following read extraction, barcode-containing reads are either mapped to a reference library of barcodes to annotate the clonal identity of a cell or processed using a reference-free approach like that employed in the population-level workflow (Figure 1). The pipeline outputs a cell metadata table containing cell ID, barcode, and number of UMIs supporting the barcode annotation, which can be imported into widely used R- and Python-based single-cell analysis packages including Seurat, SingleCellExperiment and Scanpy.

To evaluate the single-cell workflow of BARtab, we re-analyzed data from Goyal et al.'s experiment FM0-2[37] (hereafter, the FateMap dataset). Here, BRAF-mutant melanoma WM989 cells labeled with Rewind/FateMap expressed lineage barcodes were treated with vemurafenib, trametinib, a vemurafenib and trametinib combination, and an appropriate vehicle control. The published single-cell amplicon sequencing data and the list of filtered cell barcodes identified from scRNA-seq data were used as input to BARtab to annotate cells with lineage barcodes. BARtab parameters were chosen to reflect the reported FateMap dataset analysis parameters as closely as possible (see STAR Methods). Overall, BARtab could annotate lineage barcodes in almost the same total number of cells as originally reported, with an overlap of 99.6% across the four conditions (BARtab only: 12 cells; FateMap only: 145 cells; annotated by both tools: 39,884 cells). Moreover, the Pearson correlation of clone sizes in BARtab vs. FateMap ranged from 0.965 to 0.998, reinforcing the accuracy of BARtab lineage barcode annotation (Figure S5A).

BARtab applies two additional denoising steps in the single-cell workflow not found in Rewind/FateMap to eliminate PCR chimeras and UMI sequencing errors, both of which can lead to erroneous lineage barcode annotations in cells (see STAR Methods). Given that most cells in the FateMap dataset should contain a single lineage barcode following transduction of lineage barcodes at low multiplicity of infection,[30] we next evaluated the ability of BARtab to remove spurious barcode annotations in cells by calculating the number of cells annotated with just one barcode across a range of UMI count thresholds. While Goyal et al. applied a 15 UMI count threshold to reduce background lineage barcode annotations and maximize the number of cells containing a single lineage barcode,[30] we find that BARtab can annotate an equivalent number of cells at a UMI threshold of 5, demonstrating the impact of PCR chimera and UMI error correction steps in BARtab on lineage barcode annotation efficiency (Figure S5B).

To further demonstrate the single-cell annotation and analysis capabilities of BARtab and bartools, we applied the BARtab single-cell workflow to a 10× Genomics 3′ scRNA-seq dataset consisting of 14,086 SPLINTR-barcoded murine AML cells cultured *in vitro* (hereafter, the single-cell dataset).[9] Following cell QC filtering and doublet identification and removal, most cells (90%) annotated by BARtab contained a single lineage barcode consistent with our previous results[9] (Figure 4A). Of the cells that contained two or more barcodes, the majority (55.5%, 337 cells) belonged to clones represented by more than one cell, which is indicative of two independent viral barcode transduction events into the same progenitor cell. Examining the raw dataset, we observed a positive relationship between total UMI counts or total detected features per cell and lineage barcode detection status suggesting that annotation of lineage barcodes could also assist with QC of single-cell datasets (Figure 4B). We reasoned that lineage barcode annotations could also be useful to diagnose biases related to barcode delivery or recovery in subpopulations of cells within a sample. We examined the percentage lineage barcode detection relative to the median UMIs detected per cluster. This analysis revealed no overt bias in the detection of barcode annotated cells across Louvain clusters by uniform manifold approximation and projection (UMAP) visualization (Figure 4C). However, we did note that clusters with higher median transcript abundance in the single-cell dataset showed an increased percentage of lineage barcode annotated cells, supporting the positive relationship between total UMI counts and lineage barcode detection (Figure 4D).

The inclusion of lineage barcode information in a single-cell dataset can reveal distinct properties of individual clones, which can be related to phenotypic data. bartools contains several functions to assess such clone-level properties by leveraging widely used single-cell data structures within the R ecosystem (e.g., Seurat or SingleCellExperiment class objects). A simple yet powerful visualization is the number of cells detected per barcode, which we show for the single-cell dataset using the plotCellsPerGroup() function. For the single-cell dataset, this analysis revealed 16 clones represented by at least 100 cells, some of which were characterized by multiple barcode integration events (Figure 4E). In addition, using the plotMetrics() function, we could identify differences between clones for relevant

CellPress
OPEN ACCESS

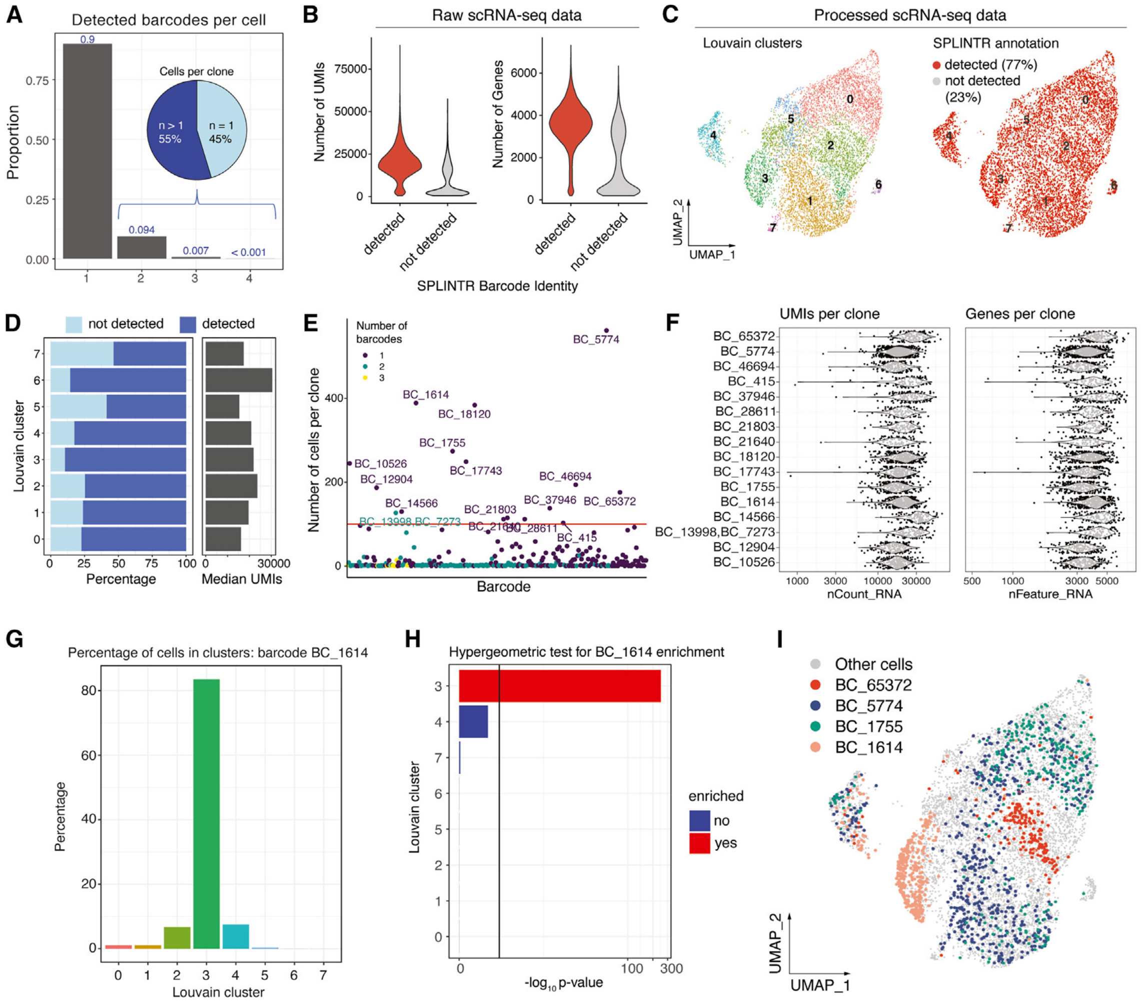

Figure 4. Expressed cellular barcode pre-processing and analysis with BARtab and bartools

(A) Histogram showing detected barcodes per cell in the single-cell dataset. Inset: pie chart showing the percentage of cells containing two or more barcodes that belong to clones represented by either a single cell ($n = 1$) or more than one cell ($n > 1$). n = number of cells.

(B) Number of UMIs detected per cell and number of genes detected per cell are shown for cells with a barcode detected (red) or not detected (gray).

(C) UMAP visualizations of the single-cell dataset following QC, filtering, and normalization showing Louvain clusters (left) and expressed barcode detection status (right).

(D) Left: percentage of cells with a lineage barcode detected per 100 cells for each Louvain cluster. Right: median UMIs per cell for each Louvain cluster.

(E) Dot plot showing the number of cells per lineage barcode. Clones comprising more than one lineage barcode are indicated.

(F) Violin plots of number of UMI counts per clone (nCount_RNA) and number of genes detected per clone (nFeature_RNA) for the 16 clones represented by 100 or more cells in the single-cell dataset.

(G) Histogram showing the percentage of cells comprising an exemplar clone, BC_1614, within each Louvain cluster.

(H) Hypergeometric test results for enrichment of an exemplar clone, BC_1614, across Louvain clusters. Vertical line indicates $-\log10\ p$ value = 2.

(I) UMAP visualization of the single-cell dataset with cells from selected clones highlighted.

metrics such as the number of individual transcripts (UMIs) or features detected for each cell (Figure 4F). These analyses can help inform on functional or phenotypic differences between individual lineages.

The degree of transcriptional homo- or heterogeneity within clonal lineages is also of interest to many studies. bartools incorporates percentage-based (Figure 4G) and hypergeometric testing approaches at the cluster level via the plotCellsInClusters() and plotClusterEnrichment() functions to determine if certain lineages are enriched within different regions of transcriptional space. This analysis revealed distinct transcriptional patterns between the top 10 most represented clones, with some clones, such as BC_1614, localizing primarily to a single Louvain cluster (Figures 4H and 4I), suggesting transcriptional conservation in these clones despite an extended period of expansion in culture. Importantly, the plotClusterEnrichment() function is agnostic to the metadata variable and can be applied to any grouping of cells desired by the user. To demonstrate this, we analyzed the enrichment of cells in different phases of the cell cycle per cluster. Cell cycle phase was annotated for the single-cell

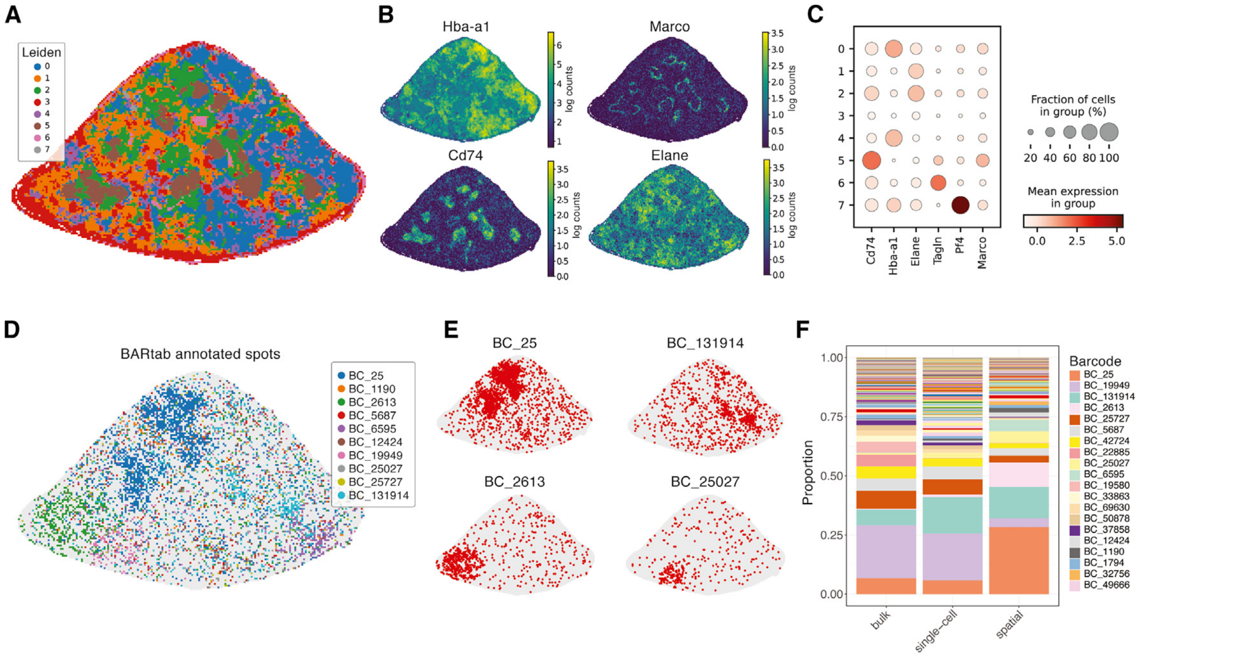

Figure 5. BARtab enables clonally resolved spatial transcriptomics

(A) Spatial section map showing Leiden clustering of the spatial dataset at resolution = 0.7.

(B) Expression pattern of marker genes corresponding to known spleen morphological regions including red pulp (*Hba-a1*), white pulp (*Cd74*), marginal zone (*Marco*), and a myeloid marker (*Elane*).

(C) Dot plot showing average expression of marker genes per Leiden cluster.

(D) Spatial section map showing spatial coordinates annotated to the top 10 most abundant lineage barcodes annotated by BARtab.

(E) Spatial section map highlighting spatial coordinates occupied by four of the top 10 most abundant lineage barcodes.

(F) Stacked bar plot showing the proportion of clones within the spatial dataset and matched population- and single-cell-level datasets. Most abundant clones are labeled.

dataset using Seurat v.4, and the enrichment of cells in G1, G2M, and S phase was analyzed using plotClusterEnrichment() in bartools. This analysis revealed that the Louvain clusters overrepresented for actively growing/dividing cells (G2M/S phase) were also overrepresented for the most abundant clones (Figure S6). Overall, these QC and analysis capabilities afforded by bartools can provide valuable insight into the nature of functional differences between different clonal lineages in a scRNA-seq dataset as directed by genetic and/or non-genetic factors particular to biological system in question.

Extending the cellular barcoding toolbox to spatial transcriptomics datasets

Recent advances in spatial genomics technologies have enabled the identification and sequencing of the endogenous spatial arrangement of individual cells *in situ.* 10× Genomics Visium V1 and BGI Stereo-seq[44] are two recently developed spatial transcriptomics technologies that utilize an oligo-dT-based transcript capture strategy like most major scRNA-seq workflows[45] and so are also compatible with 3′ expressed cellular barcoding methods to enable clonally resolved spatial transcriptomics. Briefly, both BGI Stereo-seq and 10× Genomics Visium V1 use a grid of coordinate-barcode-labeled oligonucleotides printed onto a slide in place of individual cell barcodes linked to gel emulsion beads. Like the capture of lineage-barcoded transcripts, this assay setup allows barcode-containing reads to be captured by spatial-coordinate-labeled oligonucleotides and sequenced using high-throughput methods, thus linking spatial information with clonal identity.

As a proof of concept of this approach, we applied the BGI Stereo-seq strategy to a mouse spleen sample containing SPLINTR-barcoded AML cells (hereafter, the spatial dataset).[9] The resulting BGI Stereo-seq data revealed expected splenic morphology (Figure 5A) with major Leiden cluster markers revealing the white pulp (*Cd74*), red pulp (*Hba-a1*), and marginal zone (*Marco*) regions (Figures 5B and 5C). To annotate spatial locations of the mouse spleen with lineage barcode information, we applied the BARtab single-cell workflow to the spatial dataset, specifying the “SAW” (Stereo-seq analysis workflow) aligner using the –pipeline parameter. The SAW is a pre-processing pipeline developed by BGI for processing Stereo-seq datasets that links coordinate ID and UMI information to individual transcripts.[46] Paired spatial coordinate-lineage barcode information was then imported into Scanpy for further analysis. Visualization of the top 10 most abundant clones (by total number of detected spatial coordinates) in the dataset revealed a restricted pattern of leukemic clonal outgrowth (Figure 5D), with each clone occupying a distinct spatial territory within the spleen section (Figure 5E). These different territories occupied by individual clones could simply reflect sites of initial engraftment and expansion or

different tissue microenvironments that preferentially support the outgrowth of certain clones. From a technical standpoint, it is important to note that when the distribution of clones within tissues is not uniform, clone representation within individual sections may not reflect the overall clonal representation within a tissue due to positional sampling biases. To assess this, we next compared the frequency of lineage barcodes identified in the spatial dataset to matched population-level and single-cell-level datasets previously generated from the same mouse spleen.[9] Of the top 10 most abundant clones in the spatial dataset, all were present in the matched population-level data, and 9/10 were present in the single-cell-level data. However, several clones differed in abundance (e.g., BC_25 and BC_19949), potentially due to the limited sampling of the tissue in the spatial dataset (Figure 5F). The fact that the most abundant AML clones present within matched datasets were also observed in our spatial dataset (which samples only a cross-section of the tissue) supports the utility of deriving lineage barcode annotations in spatial transcriptomics datasets using the BARtab pipeline.

DISCUSSION

Cellular barcoding approaches are widely used in biological research and will increase in utility as techniques that combine clonal lineage with other cellular modalities such as chromatin accessibility, cell surface protein expression, and spatial transcriptomics/epigenomics (e.g., histone modifications and DNA methylation) become more widely available.[47] These advanced techniques will require the application of robust data analysis tools that can be easily adapted to suit a range of experimental approaches and barcoding systems and can integrate well with current gold-standard analytical frameworks. The combined workflow provided by BARtab and bartools solves these data analysis challenges for the field by integrating the pre-processing, QC and visualization of cellular barcoding datasets into a readily accessible and flexible workflow that can accommodate many published barcode designs and experimental approaches. The BARtab/bartools framework can also be applied to perform pre-processing and QC of other conceptually similar experimental approaches such as bulk and single-cell CRISPR screens, whereby cells are effectively labeled with barcodes in the form of single guide RNAs.

Recent developments in the lineage-tracing field involve the labeling of cell lineages using CRISPR-based evolving barcodes and subsequent reconstruction of cellular lineages at the endpoint of the experiment.[48–53]. These retrospective lineage-tracing techniques are powerful in their ability to produce highly detailed phylogenetic trees, yet the significant technical and computational complexity involved with these methods has necessitated the development of dedicated software to perform lineage reconstruction such as Cassiopeia and LinRace.[54,55] While BARtab and bartools were not designed to officially support these evolving barcode approaches, future developments will further enhance the BARtab and bartools framework to support additional lineage-tracing strategies that utilize static cellular barcodes and recent sequencing-based spatial ‘omics technologies[14] to better realize the potential of combining lineage information with the rapidly expanding fields of single-cell and spatial genomics.

Limitations of the study

There are a wide range of reported methods for cellular barcoding. Many of these utilize static barcodes, in which a barcode remains unaltered from the point that it is introduced into a cell. Conversely, multiple evolving barcode methods are now available in which individual barcodes within cells can be altered over time through CRISPR-Cas9 or Cre recombinase activity, enabling the retrospective reconstruction of higher-resolution lineage trees. BARtab and bartools are written primarily to handle data originating from static cellular barcoding methodologies and currently do not officially support evolving barcode methods, although several functions already available within BARtab and bartools will likely have utility for data arising from these experimental approaches. BARtab was designed as a flexible pipeline allowing extraction and filtering of barcodes from different barcoding systems and kinds of high-throughput sequencing data. However, benchmarking BARtab against similar tools was difficult due to the lack of a gold-standard dataset with known barcode abundance and differing parameter flexibility between tools. We benchmarked BARtab by reproducing published results, showing improved runtime and sensitivity. Benchmarking on simulated data could give further insight into accuracy and optimal parameter selection.

STAR★METHODS

Detailed methods are provided in the online version of this paper and include the following:

- KEY RESOURCES TABLE
- RESOURCE AVAILABILITY
  - Lead contact
  - Materials availability
  - Data and code availability
- METHOD DETAILS
  - Tissue culture
  - Dose escalation experiments and analysis
  - Single cell dataset capture and analysis
  - Spatial transcriptomics capture and analysis
  - Software availability
- QUANTIFICATION AND STATISTICAL ANALYSIS
  - BARtab workflow implementation
  - BARtab population-level workflow
  - BARtab single-cell workflow
  - BARtab performance comparison – Population level data
  - BARtab performance comparison – Single-cell data

SUPPLEMENTAL INFORMATION

Supplemental information can be found online at https://doi.org/10.1016/j.crmeth.2024.100763.

ACKNOWLEDGMENTS

The authors would like to acknowledge the support of the Molecular Genomics and Flow Cytometry core facilities at the Peter MacCallum Cancer Centre, Melbourne, Australia, for assistance with single-cell sample preparation and sequencing; BGI Research, Shenzhen, China, and BGI Australia for assistance with Stereo-seq library generation, sequencing, and bioinformatics support; and the Oshlack group at the Peter MacCallum Cancer Centre for helpful

discussions. The authors also thank the following funders for fellowship, scholarship, and grant support: Leukemia and Lymphoma Society Career Development Fellowship #3411-22, Gilead Sciences International Research Scholarship, NHMRC Ideas Grant #2028298 (D.V.), Cancer Council Victoria Sir Edward Dunlop Research Fellowship, NHMRC Investigator Grant #1196749, Howard Hughes Medical Institute International Research Scholarship #55008729 (M.A.D.), and NHMRC Project Grants #1085015, #1106444, and #1128984 (M.A.D.).

AUTHOR CONTRIBUTIONS

D.V. and H.H. designed and wrote the BARtab and bartools software with assistance from E.Y.L. and L.T. D.V., H.H., L.T., and E.Y.L. tested the software and assisted with the development of best-practices workflows and analysis vignettes for BARtab and bartools. K.A.F. performed experimental work for the dose-escalation and single-cell datasets. D.V., H.H., and L.T. wrote the manuscript with helpful contributions from all other authors. The project was supervised by D.V. and M.A.D. All authors read and approved the manuscript for publication.

DECLARATION OF INTERESTS

M.A.D. has been a member of advisory boards for GSK, CTX CRC, Storm Therapeutics, Celgene, and Cambridge Epigenetix. The Dawson laboratory is a recipient of grant funding from the emerging sciences fund administered through Pfizer.

Received: December 20, 2023
Revised: February 25, 2024
Accepted: April 3, 2024
Published: April 25, 2024

REFERENCES

1. Serrano, A., Berthelet, J., Naik, S.H., and Merino, D. (2022). Mastering the use of cellular barcoding to explore cancer heterogeneity. Nat. Rev. Cancer *22*, 609–624. https://doi.org/10.1038/s41568-022-00500-2.
2. Naik, S.H., Schumacher, T.N., and Perié, L. (2014). Cellular barcoding: a technical appraisal. Exp. Hematol. *42*, 598–608. https://doi.org/10.1016/j.exphem.2014.05.003.
3. Kebschull, J.M., and Zador, A.M. (2018). Cellular barcoding: lineage tracing, screening and beyond. Nat. Methods *15*, 871–879. https://doi.org/10.1038/s41592-018-0185-x.
4. Baron, C.S., and van Oudenaarden, A. (2019). Unravelling cellular relationships during development and regeneration using genetic lineage tracing. Nat. Rev. Mol. Cell Biol. *20*, 753–765. https://doi.org/10.1038/s41580-019-0186-3.
5. Lu, R., Neff, N.F., Quake, S.R., and Weissman, I.L. (2011). Tracking single hematopoietic stem cells in vivo using high-throughput sequencing in conjunction with viral genetic barcoding. Nat. Biotechnol. *29*, 928–933. https://doi.org/10.1038/nbt.1977.
6. Bramlett, C., Jiang, D., Nogalska, A., Eerdeng, J., Contreras, J., and Lu, R. (2020). Clonal tracking using embedded viral barcoding and high-throughput sequencing. Nat. Protoc. *15*, 1436–1458. https://doi.org/10.1038/s41596-019-0290-z.
7. Biddy, B.A., Kong, W., Kamimoto, K., Guo, C., Waye, S.E., Sun, T., and Morris, S.A. (2018). Single-cell mapping of lineage and identity in direct reprogramming. Nature *564*, 219–224. https://doi.org/10.1038/s41586-018-0744-4.
8. Weinreb, C., Rodriguez-Fraticelli, A., Camargo, F.D., and Klein, A.M. (2020). Lineage tracing on transcriptional landscapes links state to fate during differentiation. Sci New York N Y *367*, eaaw3381. https://doi.org/10.1126/science.aaw3381.
9. Fennell, K.A., Vassiliadis, D., Lam, E.Y.N., Martelotto, L.G., Balic, J.J., Hollizeck, S., Weber, T.S., Semple, T., Wang, Q., Miles, D.C., et al. (2022). Non-genetic determinants of malignant clonal fitness at single-cell resolution. Nature *601*, 125–131. https://doi.org/10.1038/s41586-021-04206-7.
10. Oren, Y., Tsabar, M., Cuoco, M.S., Amir-Zilberstein, L., Cabanos, H.F., Hütter, J.C., Hu, B., Thakore, P.I., Tabaka, M., Fulco, C.P., et al. (2021). Cycling cancer persister cells arise from lineages with distinct programs. Nature *596*, 576–582. https://doi.org/10.1038/s41586-021-03796-6.
11. Chang, M.T., Shanahan, F., Nguyen, T.T.T., Staben, S.T., Gazzard, L., Yamazoe, S., Wertz, I.E., Piskol, R., Yang, Y.A., Modrusan, Z., et al. (2022). Identifying transcriptional programs underlying cancer drug response with TraCe-seq. Nat. Biotechnol. *40*, 86–93. https://doi.org/10.1038/s41587-021-01005-3.
12. Eyler, C.E., Matsunaga, H., Hovestadt, V., Vantine, S.J., van Galen, P., and Bernstein, B.E. (2020). Single-cell lineage analysis reveals genetic and epigenetic interplay in glioblastoma drug resistance. Genome Biol. *21*, 174. https://doi.org/10.1186/s13059-020-02085-1.
13. Emert, B.L., Cote, C.J., Torre, E.A., Dardani, I.P., Jiang, C.L., Jain, N., Shaffer, S.M., and Raj, A. (2021). Variability within rare cell states enables multiple paths toward drug resistance. Nat. Biotechnol. *39*, 865–876. https://doi.org/10.1038/s41587-021-00837-3.
14. Ratz, M., von Berlin, L., Larsson, L., Martin, M., Westholm, J.O., La Manno, G., Lundeberg, J., and Frisén, J. (2022). Clonal relations in the mouse brain revealed by single-cell and spatial transcriptomics. Nat. Neurosci. *25*, 285–294. https://doi.org/10.1038/s41593-022-01011-x.
15. Lyne, A.-M., Kent, D.G., Laurenti, E., Cornils, K., Glauche, I., and Perié, L. (2018). A Track of the Clones: New developments in cellular barcoding. Exp. Hematol. *68*, 15–20. https://doi.org/10.1016/j.exphem.2018.11.005.
16. Thielecke, L., Cornils, K., and Glauche, I. (2020). genBaRcode: a comprehensive R-package for genetic barcode analysis. Bioinformatics *36*, 2189–2194. https://doi.org/10.1093/bioinformatics/btz872.
17. Espinoza, D.A., Mortlock, R.D., Koelle, S.J., Wu, C., and Dunbar, C.E. (2021). Interrogation of clonal tracking data using barcodetrackR. Nat. Comput. Sci. *1*, 280–289. https://doi.org/10.1038/s43588-021-00057-4.
18. Hadj Abed, L., Tak, T., Cosgrove, J., and Perié, L. (2022). CellDestiny: A RShiny application for the visualization and analysis of single-cell lineage tracing data. Front. Med. *9*, 919345. https://doi.org/10.3389/fmed.2022.919345.
19. Rodriguez-Fraticelli, A.E., Weinreb, C., Wang, S.-W., Migueles, R.P., Jankovic, M., Usart, M., Klein, A.M., Lowell, S., and Camargo, F.D. (2020). Single-cell lineage tracing unveils a role for TCF15 in haematopoiesis. Nature *583*, 585–589. https://doi.org/10.1038/s41586-020-2503-6.
20. Gardner, A., Morgan, D., Al'Khafaji, A., and Brock, A. (2022). Biomedical Engineering Technologies, Volume 2 - Functionalized Lineage Tracing for the Study and Manipulation of Heterogeneous Cell Populations. Methods Mol. Biol. *2394*, 109–131. https://doi.org/10.1007/978-1-0716-1811-0_8.
21. Gutierrez, C., Al'Khafaji, A.M., Brenner, E., Johnson, K.E., Gohil, S.H., Lin, Z., Knisbacher, B.A., Durrett, R.E., Li, S., Parvin, S., et al. (2021). Multifunctional barcoding with ClonMapper enables high-resolution study of clonal dynamics during tumor evolution and treatment. Nat. Can. (Ott.) *2*, 758–772. https://doi.org/10.1038/s43018-021-00222-8.
22. NKI-GCF (2023). Xcalibr - eXtracting Counting and LInking to Barcode References.
23. Di Tommaso, P., Chatzou, M., Floden, E.W., Barja, P.P., Palumbo, E., and Notredame, C. (2017). Nextflow enables reproducible computational workflows. Nat. Biotechnol. *35*, 316–319. https://doi.org/10.1038/nbt.3820.
24. Zorita, E., Cuscó, P., and Filion, G.J. (2015). Starcode: sequence clustering based on all-pairs search. Bioinformatics *31*, 1913–1919. https://doi.org/10.1093/bioinformatics/btv053.
25. Langmead, B., Trapnell, C., Pop, M., and Salzberg, S.L. (2009). Ultrafast and memory-efficient alignment of short DNA sequences to the human genome. Genome Biol. *10*, R25. https://doi.org/10.1186/gb-2009-10-3-r25.

CellPress
OPEN ACCESS

26. Magoč, T., and Salzberg, S.L. (2011). FLASH: fast length adjustment of short reads to improve genome assemblies. Bioinformatics *27*, 2957–2963. https://doi.org/10.1093/bioinformatics/btr507.

27. Danecek, P., Bonfield, J.K., Liddle, J., Marshall, J., Ohan, V., Pollard, M.O., Whitwham, A., Keane, T., McCarthy, S.A., Davies, R.M., and Li, H. (2021). Twelve years of SAMtools and BCFtools. GigaScience *10*, giab008. https://doi.org/10.1093/gigascience/giab008.

28. Martin, M. (2011). Cutadapt removes adapter sequences from high-throughput sequencing reads. EMBnetJ *17*, 10–12. https://doi.org/10.14806/ej.17.1.200.

29. Chen, S., Zhou, Y., Chen, Y., and Gu, J. (2018). fastp: an ultra-fast all-in-one FASTQ preprocessor. Bioinformatics *34*, i884–i890. https://doi.org/10.1093/bioinformatics/bty560.

30. Goyal, Y., Busch, G.T., Pillai, M., Li, J., Boe, R.H., Grody, E.I., Chelvanambi, M., Dardani, I.P., Emert, B., Bodkin, N., et al. (2023). Diverse clonal fates emerge upon drug treatment of homogeneous cancer cells. Nature *620*, 651–659. https://doi.org/10.1038/s41586-023-06342-8.

31. Amezquita, R.A., Lun, A.T.L., Becht, E., Carey, V.J., Carpp, L.N., Geistlinger, L., Marini, F., Rue-Albrecht, K., Risso, D., Soneson, C., et al. (2020). Orchestrating single-cell analysis with Bioconductor. Nat. Methods *17*, 137–145. https://doi.org/10.1038/s41592-019-0654-x.

32. Hao, Y., Hao, S., Andersen-Nissen, E., Mauck, W.M., Zheng, S., Butler, A., Lee, M.J., Wilk, A.J., Darby, C., Zager, M., et al. (2021). Integrated analysis of multimodal single-cell data. Cell *184*, 3573–3587.e29. https://doi.org/10.1016/j.cell.2021.04.048.

33. Wolf, F.A., Angerer, P., and Theis, F.J. (2018). SCANPY: large-scale single-cell gene expression data analysis. Genome Biol. *19*, 15. https://doi.org/10.1186/s13059-017-1382-0.

34. Robinson, M.D., McCarthy, D.J., and Smyth, G.K. (2010). edgeR: a Bioconductor package for differential expression analysis of digital gene expression data. Bioinformatics *26*, 139–140. https://doi.org/10.1093/bioinformatics/btp616.

35. Johnson, M.S., Venkataram, S., and Kryazhimskiy, S. (2022). Best Practices in Designing, Sequencing and Identifying Random DNA Barcodes. https://doi.org/10.32942/osf.io/t58xw.

36. Thielecke, L., Aranyossy, T., Dahl, A., Tiwari, R., Roeder, I., Geiger, H., Fehse, B., Glauche, I., and Cornils, K. (2017). Limitations and challenges of genetic barcode quantification. Sci. Rep. *7*, 43249. https://doi.org/10.1038/srep43249.

37. Bell, C.C., Fennell, K.A., Chan, Y.-C., Rambow, F., Yeung, M.M., Vassiliadis, D., Lara, L., Yeh, P., Martelotto, L.G., Rogiers, A., et al. (2019). Targeting enhancer switching overcomes non-genetic drug resistance in acute myeloid leukaemia. Nat. Commun. *10*, 2723. https://doi.org/10.1038/s41467-019-10652-9.

38. Robinson, M.D., and Oshlack, A. (2010). A scaling normalization method for differential expression analysis of RNA-seq data. Genome Biol. *11*, R25. https://doi.org/10.1186/gb-2010-11-3-r25.

39. Oksanen, J., Simpson, G.L., Blanchet, F.G., Kindt, R., Legendre, P., Minchin, P.R., O'Hara, R.B., Solymos, P., Stevens, M.H.H., Szoecs, E., et al. (2022). Vegan: Community Ecology Package.

40. Zeileis, A. (2014). Ineq: Measuring Inequality (Concentration, and Poverty).

41. Jindal, K., Adil, M.T., Yamaguchi, N., Yang, X., Wang, H.C., Kamimoto, K., Rivera-Gonzalez, G.C., and Morris, S.A. (2023). Single-cell lineage capture across genomic modalities with CellTag-multi reveals fate-specific gene regulatory changes. Nat. Biotechnol., 1–14. https://doi.org/10.1038/s41587-023-01931-4.

42. Zheng, G.X.Y., Terry, J.M., Belgrader, P., Ryvkin, P., Bent, Z.W., Wilson, R., Ziraldo, S.B., Wheeler, T.D., McDermott, G.P., Zhu, J., et al. (2017). Massively parallel digital transcriptional profiling of single cells. Nat. Commun. *8*, 14049. https://doi.org/10.1038/ncomms14049.

43. Kaminow, B., Yunusov, D., and Dobin, A. (2021). STARsolo: accurate, fast and versatile mapping/quantification of single-cell and single-nucleus RNA-seq data. Preprint at bioRxiv. https://doi.org/10.1101/2021.05.05.442755.

44. Chen, A., Liao, S., Cheng, M., Ma, K., Wu, L., Lai, Y., Qiu, X., Yang, J., Xu, J., Hao, S., et al. (2022). Spatiotemporal transcriptomic atlas of mouse organogenesis using DNA nanoball-patterned arrays. Cell *185*, 1777–1792.e21. https://doi.org/10.1016/j.cell.2022.04.003.

45. Tian, L., Chen, F., and Macosko, E.Z. (2023). The expanding vistas of spatial transcriptomics. Nat. Biotechnol. *41*, 773–782. https://doi.org/10.1038/s41587-022-01448-2.

46. Gong, C., Li, S., Wang, L., Zhao, F., Fang, S., Yuan, D., Zhao, Z., He, Q., Li, M., Liu, W., et al. (2023). SAW: An efficient and accurate data analysis workflow for Stereo-seq spatial transcriptomics. Preprint at bioRxiv. https://doi.org/10.1101/2023.08.20.554064.

47. Wagner, D.E., and Klein, A.M. (2020). Lineage tracing meets single-cell omics: opportunities and challenges. Nat. Rev. Genet. *21*, 410–427. https://doi.org/10.1038/s41576-020-0223-2.

48. McKenna, A., Findlay, G.M., Gagnon, J.A., Horwitz, M.S., Schier, A.F., and Shendure, J. (2016). Whole-organism lineage tracing by combinatorial and cumulative genome editing. Science *353*, aaf7907. https://doi.org/10.1126/science.aaf7907.

49. Raj, B., Wagner, D.E., McKenna, A., Pandey, S., Klein, A.M., Shendure, J., Gagnon, J.A., and Schier, A.F. (2018). Simultaneous single-cell profiling of lineages and cell types in the vertebrate brain. Nat. Biotechnol. *36*, 442–450. https://doi.org/10.1038/nbt.4103.

50. Simeonov, K.P., Byrns, C.N., Clark, M.L., Norgard, R.J., Martin, B., Stanger, B.Z., Shendure, J., McKenna, A., and Lengner, C.J. (2021). Single-cell lineage tracing of metastatic cancer reveals selection of hybrid EMT states. Cancer Cell *39*, 1150–1162.e9. https://doi.org/10.1016/j.ccell.2021.05.005.

51. Alemany, A., Florescu, M., Baron, C.S., Peterson-Maduro, J., and van Oudenaarden, A. (2018). Whole-organism clone tracing using single-cell sequencing. Nature *556*, 108–112. https://doi.org/10.1038/nature25969.

52. Bowling, S., Sritharan, D., Osorio, F.G., Nguyen, M., Cheung, P., Rodriguez-Fraticelli, A., Patel, S., Yuan, W.-C., Fujiwara, Y., Li, B.E., et al. (2020). An Engineered CRISPR-Cas9 Mouse Line for Simultaneous Readout of Lineage Histories and Gene Expression Profiles in Single Cells. Cell *181*, 1410–1422.e27. https://doi.org/10.1016/j.cell.2020.04.048.

53. Li, L., Bowling, S., McGeary, S.E., Yu, Q., Lemke, B., Alcedo, K., Jia, Y., Liu, X., Ferreira, M., Klein, A.M., et al. (2023). A mouse model with high clonal barcode diversity for joint lineage, transcriptomic, and epigenomic profiling in single cells. Cell *186*, 5183–5199.e22. https://doi.org/10.1016/j.cell.2023.09.019.

54. Pan, X., Li, H., Putta, P., and Zhang, X. (2023). LinRace: cell division history reconstruction of single cells using paired lineage barcode and gene expression data. Nat. Commun. *14*, 8388. https://doi.org/10.1038/s41467-023-44173-3.

55. Jones, M.G., Khodaverdian, A., Quinn, J.J., Chan, M.M., Hussmann, J.A., Wang, R., Xu, C., Weissman, J.S., and Yosef, N. (2020). Inference of single-cell phylogenies from lineage tracing data using Cassiopeia. Genome Biol. *21*, 92. https://doi.org/10.1186/s13059-020-02000-8.

56. Stuart, T., Butler, A., Hoffman, P., Hafemeister, C., Papalexi, E., Mauck, W.M., Hao, Y., Stoeckius, M., Smibert, P., and Satija, R. (2019). Comprehensive Integration of Single-Cell Data. Cell *177*, 1888–1902.e21. https://doi.org/10.1016/j.cell.2019.05.031.

57. Tange, O. (2023). GNU Parallel (Zenodo).

58. Ewels, P., Magnusson, M., Lundin, S., and Käller, M. (2016). MultiQC: summarize analysis results for multiple tools and samples in a single report. Bioinformatics *32*, 3047–3048. https://doi.org/10.1093/bioinformatics/btw354.

59. Fong, C.Y., Gilan, O., Lam, E.Y.N., Rubin, A.F., Ftouni, S., Tyler, D., Stanley, K., Sinha, D., Yeh, P., Morison, J., et al. (2015). BET inhibitor resistance emerges from leukaemia stem cells. Nature *525*, 538–542. https://doi.org/10.1038/nature14888.

60. Grüning, B., Grüning, B., Sjödin, A., Sjödin, A., Rowe, J., Tomkins-Tinch, C.H., Valieris, R., Köster, J., and Köster, J. (2018). Bioconda: sustainable and comprehensive software distribution for the life sciences. Nat. Methods *15*, 475–476. https://doi.org/10.1038/s41592-018-0046-7.

61. Kurtzer, G.M., Sochat, V., and Bauer, M.W. (2017). Singularity: Scientific containers for mobility of compute. PLoS One *12*, e0177459. https://doi.org/10.1371/journal.pone.0177459.

62. McGinnis, C.S., Murrow, L.M., and Gartner, Z.J. (2019). DoubletFinder: Doublet Detection in Single-Cell RNA Sequencing Data Using Artificial Nearest Neighbors. Cell Syst. *8*, 329–337.e4. https://doi.org/10.1016/j.cels.2019.03.003.

63. Xu, Z., Wang, W., Yang, T., Li, L., Ma, X., Chen, J., Wang, J., Huang, Y., Gould, J., Lu, H., et al. (2024). STOmicsDB: a comprehensive database for spatial transcriptomics data sharing, analysis and visualization. Nucleic Acids Res. *52*, D1053–D1061. https://doi.org/10.1093/nar/gkad933.

64. Fang, S., Xu, M., Cao, L., Liu, X., Bezulj, M., Tan, L., Yuan, Z., Li, Y., Xia, T., Guo, L., et al. (2023). Stereopy: modeling comparative and spatiotemporal cellular heterogeneity via multi-sample spatial transcriptomics. Preprint at bioRxiv. https://doi.org/10.1101/2023.12.04.569485.

65. Babraham Bioinformatics FastQC https://www.bioinformatics.babraham.ac.uk/projects/fastqc/

66. Smith, T., Heger, A., and Sudbery, I. (2017). UMI-tools: modeling sequencing errors in Unique Molecular Identifiers to improve quantification accuracy. Genome Res. *27*, 491–499. https://doi.org/10.1101/gr.209601.116.

CellPress
OPEN ACCESS

STAR★METHODS

KEY RESOURCES TABLE

REAGENT or RESOURCE	SOURCE	IDENTIFIER
**Biological samples**		
Mouse: MLL-AF9 cell samples	This study	N/A
Mouse: C57BL/6J spleen samples	Fennell & Vassiliadis et al. 2022[9]	N/A
**Chemicals, peptides, and recombinant proteins**		
Dimethyl Sulfoxide (DMSO)	Merck	D4540
IBET-151 (GSK1210151A)	Selleckchem	S2780
Cytarabine (AraC)	Merck	PHR1787
Recombinant mouse IL-3	PeproTech	213–13
Recombinant human IL-6	PeproTech	200–06
Recombinant mouse SCF	PeproTech	250–03
Viagen DirectPCR cell lysis reagent	Viagen Biotech	301-C
Proteinase K (20 mg/mL)	Qiagen	RP107B-1
Polybrene	Merck	TR-1003-G
Polyethylenimine, Linear (PEI)	Polysciences Inc.	23966–1
**Critical commercial assays**		
10x Genomics Chromium Next GEM Single Cell 3' Kit v3.1	10x Genomics	PN-1000268
BGI Stereo-seq kit	BGI Research	V1.0
**Deposited data**		
Raw and analyzed data	This study	GEO: GSE246611
Processed stereo-seq spatial dataset at different bin sizes	This study	https://zenodo.org/records/10685805
Goyal et al. 2023 Nature single-cell lineage barcode data (FM0-2 sample)	Goyal et al. 2023 Nature[30]	https://figshare.com/articles/dataset/FateMap_Paper_datasets_3_Goyal_et_al_2021_Biorxiv_/22806494
Goyal et al. 2023 Nature single-cell cell barcode data (FM0-2 sample)	Goyal et al. 2023 Nature[30]	GEO: GSM7434409, GSM7434410, GSM7434411, GSM7434412
Fennel et al. 2022 Nature MLL-AF9 barcode-seq data	Fennell & Vassiliadis et al. 2022[9]	GEO: GSM4912515
Fennel et al. 2022 Nature MLL-AF9 single-cell RNA-seq data	Fennell & Vassiliadis et al. 2022[9]	GEO: GSM4912551
**Experimental models: Cell lines**		
Mouse: MLL-AF9	Fennell & Vassiliadis et al. 2022[9]	N/A
**Experimental models: Organisms/strains**		
Mouse: C57BL/6J	The Jackson Laboratory	000664
Mouse: B6.SJL-Ptprc^{a} Pepc^{b}/BoyJ	The Jackson Laboratory	002014
**Oligonucleotides**		
Population based DNA barcode-seq library oligos	Fennell & Vassiliadis et al. 2022[9]	https://www.addgene.org/pooled-library/dawson-splintr-libraries/
**Recombinant DNA**		
SPLINTR mCHERRY version 0 lentiviral barcode library	Fennell & Vassiliadis et al. 2022[9]	N/A
SPLINTR BFP version 1 lentiviral barcode library	Fennell & Vassiliadis et al. 2022[9]	Addgene #179776
**Software and algorithms**		
Code repository for the study	This study	https://zenodo.org/doi/10.5281/zenodo.10896642

(*Continued on next page*)

Continued		
REAGENT or RESOURCE	SOURCE	IDENTIFIER
Code for BARtab benchmarking studies	This study	https://zenodo.org/doi/10.5281/zenodo.10199565
BARtab pipeline v1.4	This study	https://zenodo.org/doi/10.5281/zenodo.10896646
Bartools package v1.0.0	This study	https://zenodo.org/doi/10.5281/zenodo.10896648
Nextflow v23.04.1	Sequera Labs	https://www.nextflow.io/
R v4.2	The R Foundation	https://www.r-project.org/
Cell Ranger v.3.1.0	10x Genomics	https://www.10xgenomics.com/support/software/cell-ranger/latest
Goyal et al. 2023 Nature code repository	Goyal et al. 2023 Nature[30]	https://zenodo.org/records/8000328
Rewind/FateMap codebase (commit # 9b8c24f)	Goyal et al. 2023 Nature[30]	https://github.com/arjunrajlaboratory/timemachine
pycashier v23.1.2	Gutierrez, C. et al. 2021. Nature Cancer[21]	https://pypi.org/project/pycashier/23.1.2/
ImageStudio v2.0.1	BGI	https://en.stomics.tech/products/stomics-software/stomics-offline-software/list.html
StereoMap v2.1.0	BGI	https://en.stomics.tech/products/stomics-software/stomics-offline-software/list.html
Stereopy v0.11.0	BGI	https://github.com/STOmics/Stereopy
SAW v6.1.0	BGI	https://github.com/STOmics/SAW
Scanpy v1.9.3	Wolf, F. A. et al. Genome Biol 2018[33]	https://github.com/scverse/scanpy
Seurat v4	Stuart, T. et al. Cell 2019[56]	https://cran.r-project.org/web/packages/Seurat/index.html
Other		
RPMI-1640	Gibco	21875034
Fetal bovine serum	Gibco	26140079
Penicillin-Streptomycin (10,000 U/mL)	Thermo Fisher Scientific	15140122
GlutaMAX	Thermo Fisher Scientific	35050061

RESOURCE AVAILABILITY

Lead contact

Requests for protocols and reagents can be directed to Dane Vassiliadis (dane.vassiliadis@petermac.org).

Materials availability

The SPLINTR V1 lineage barcode libraries used in this study are available from Addgene (Pooled Library #179774, #179775, #179776).

All other materials used in this study are commercially available.

Data and code availability

- The dose escalation, single-cell and spatial datasets have been deposited at the NCBI Gene Expression Omnibus (accession #GSE246611). We have uploaded the processed stereo-seq spatial dataset at different bin sizes to Zenodo to facilitate further methods development for clonally resolved spatial transcriptomics data (https://zenodo.org/records/10685805). Code to reproduce the analyses in this manuscript can be found at GitHub (https://github.com/DaneVass/bartools_manuscript_code). The BARtab documentation (https://github.com/DaneVass/BARtab/blob/main/README.md) contains information on pipeline installation and execution. The bartools documentation (https://danevass.github.io/bartools/) contains further worked examples of cellular barcoding analysis from other previously published datasets, and describes workflows for reference library construction, single cell RNA-seq sample QC, annotation, and analysis. Results of the BARtab performance comparisons are deposited on Zenodo https://zenodo.org/records/10685739.

- BARtab and bartools are both freely available at https://github.com/DaneVass/bartools and https://github.com/DaneVass/BARtab under MIT and GPL3 licenses respectively. Archival DOIs are listed in the key resources table. Extensive documentation is available at https://danevass.github.io/bartools. Bartools can be installed into R v3.5 or above using instructions that can be found at https://github.com/DaneVass/bartools. Bartools integrates with other packages available from the Bioconductor project and utilises functions and object classes from edgeR [34], ineq [41] and vegan [40]. Graphical functions from base R and ggplot2 are also utilised within BARtab and bartools.
- BARtab can be installed into macOS or UNIX environments compatible with the Nextflow workflow manager v23.04 and later.[23] BARtab depends on common bioinformatic tools including Samtools,[27] Bowtie,[25] Starcode,[24] FLASh,[26] FastQC,[57] fastp,[29] UMI-tools,[58] MultiQC,[59] and GNU parallel,[60] and requires Python v3.8 or greater. We provide a Docker image compatible with Singularity to facilitate pipeline portability across systems. All dependencies required to successfully run the pipeline are also available from the conda and bioconda projects.[60] Available parameters for BARtab are specified in the documentation (https://github.com/DaneVass/BARtab/blob/main/README.md) which also details approaches for pipeline and software dependency installation via Singularity,[61] Docker or conda environments.
- Any additional information required to reanalyze the data reported in this paper is available from the lead contact upon request.

METHOD DETAILS

Tissue culture

Female mouse MLL-AF9 leukemia cells were obtained from previous studies[9,59] and cultured in RPMI-1640 medium supplemented with mouse IL-3 (10 ng mL^{-1}), human IL-6 (10 ng mL^{-1}), mouse SCF (50 ng mL^{-1}) 20% Fetal bovine serum, streptomycin (100 μg mL^{-1}), penicillin (100 U ml^{-1}) and 2 mM GlutaMAX (Thermo Fisher Scientific) in 5% CO_2 at 37°C. Cell lines were routinely tested for mycoplasma by the Peter MacCallum Genotyping Core Facility and confirmed negative for the duration of the study.

Dose escalation experiments and analysis

Five hundred thousand mouse MLL-AF9 cells were transduced in polybrene (8.5 mg/mL) by spin infection (90 min at 1,250 r.cf.) with lentivirus prepared from the SPLINTR BFP barcode library (Addgene #179776) at a low MOI to ensure single copy integration into cells.[9] BFP positive cells were sorted using a FACS Aria Fusion flow sorter 3 (BD Biosciences) flow cytometer 48 h after transduction. Approximately $5x10^4$ mCherry positive cells were seeded into liquid culture and expanded for seven days. Following expansion, $5x10^5$ cells were harvested as a baseline timepoint zero (T_0) sample, lysed in 40uL Viagen DirectPCR cell lysis reagent containing 0.5 mg/mL Proteinase K (Qiagen) and processed for population based SPLINTR barcode sequencing as described previously.[9] To maintain a minimum 20-fold representation of the original theoretical maximum of $5x10^4$ total barcodes per treatment arm, $1x10^6$ cells per replicate and treatment condition were seeded into liquid culture. For the dose escalation arms of the experiment, cultures were supplemented with either 0.1% v/v DMSO, 400nM IBET or 300nM AraC. For the high dose arms, liquid cultures were supplemented with 800nM IBET or 700nM AraC which we had previously determined to represent the equivalent of an IC90 dose in this cell line (data not shown). Drugs were replenished every three days by pelleting the cells at 400 rcf for 5 min at 37°C, and replating in fresh medium with drug. For the dose escalation arms, every 7 days, 5×10^5 cells were replated into fresh medium supplemented with an increased concentration of IBET (TP1 - 400nM, TP-2 - 600nM, TP-3 – 800nM and TP-4 - 1000nM), AraC (TP1 - 300nM, TP-2 - 300nM, TP-3 – 300nM and TP-4 - 500nM) or maintained in 0.1% v/v DMSO. Per timepoint, 1 million cells from each biological replicate were harvested and processed as above for population based SPLINTR barcode sequencing.[9]

Raw sequence data from population-based dose-escalation were processed using BARtab v1.4 with the following parameters: mode: “single-bulk”, upconstant: “CGATTGACTA”, downconstant: “TGCTAATGCG”, alnmismatches: 1, minqual: 20, pctqual: 80, constants: “up”, constantmismatches: 0.1, barcode_length: 60. Count files were imported into R v4.2 and further analyzed with bartools v1.0.0.

Single cell dataset capture and analysis

$5x10^5$ mouse MLL-AF9 cells were transduced with the SPLINTR V0 mCherry barcode library (Table S2). Fluorochrome positive cells were isolated using a BD Fusion 5 flow sorter 48 h after transduction and expanded for seven days in liquid culture as described above. Cells were then processed for single cell transcriptomic capture using the 10x Genomics 3′ V3 single cell RNA-seq platform. Count matrices were generated from demultiplexed scRNA-seq fastq files using the 10x Genomics Cell Ranger (v.3.1.0) count pipeline against the mm10/GRCm38 reference genome. Quality control was performed using Seurat v4 in R v4.2.[62] Low-quality cells were removed by filtering out cells that had between 2000 and 5000 detected genes and fewer than 40,000 unique molecular identifiers (UMI). Cells with greater than 10% mitochondrial RNA content were also removed. Doublets were predicted using DoubletFinder[62] assuming 10% doublets. For lineage barcode identification and annotation to cells, unmapped reads in BAM file format were extracted from the dataset using Samtools (v1.9) and used as input for BARtab v1.4 running in “single-cell” mode with parameters as follows: mode “single-cell” pipeline “cellranger”, upconstant: “CGATTGACTA”, downconstant: “TGCTAATGCG”, constants “all”. Downstream analysis was performed with Seurat v4 and bartools v1.0.0.

Spatial transcriptomics capture and analysis

Mouse spleen samples containing SPLINTR barcoded MLL-AF9 leukemia cells flash frozen in liquid nitrogen were obtained from a previous study.[9] BGI Stereo-seq assays were performed using version 1.0 of the Stereo-seq kit and sequenced by BGI genomics group in Shenzhen, China. Section image data was quality controlled with ImageStudio v2.0.1 (BGI). Count matrices were generated from demultiplexed Stereo-seq fastq files using SAW v6.1.0. Reads were aligned to the mm10/GRCm38 reference genome. The Stereo-seq data was manually segmented using the lasso function of StereoMap v2.1.0 (BGI).[63] Stereopy v0.11.0 (BGI)[64] was used to aggregate the count matrix to bin50. Bins with less than 600 UMI were removed, counts were log transformed and highly variable genes were identified using default parameters in Scanpy v1.9.3.[33] Downstream data scaling, PCA and UMAP dimensionality reduction were performed using default parameters. Leiden clustering was performed using resolution = 0.7.

To annotate spatial coordinates with lineage barcodes, unaligned reads resulting from the SAW pipeline were processed with BARtab v1.4 using default parameters except for the following: upconstant: "TGACCATGTACGATTGACTA", downconstant: "TGCTAATGCGTACTGACTAG", constants: “both”, constantmismatches: 0.2, barcode_length: 60, mode: "single-cell", input_type: "fastq", pipeline: "saw". Barcodes were aligned to the SPLINTR GFP reference library.[9] Barcode counts were aggregated to bins and merged with the spatial dataset count matrix based on coordinate ID. To compare clonal composition in population-level, single-cell and spatial data, we used published DNA barcode-seq and scRNA-seq data of the same mouse spleen (GEO: GSM4912416, GSM4912482). BARtab v1.4 was run on DNA barcode-seq data with default parameters except the following: mode: “single-bulk”, upconstant: "TGACCATGTACGATTGACTA", up_coverage: 20, constants: "up”, constantmismatches: 20, barcode_length: 60, min_readlength: 43. Barcode reads were aligned to the SPLINTR GFP reference library.[9] Barcodes present in both PCR replicates with at least 5 reads were retained and read counts were averaged. Matched scRNA-seq data was obtained from a previous study.[9] BARtab was run using unaligned reads from the Cell Ranger scRNA-seq alignment to the mm10 reference genome in BAM format as input. Default parameters were used except the following: mode: “single cell”, input_type: “bam”, upconstant: "TGACCATGTAC GATTGACTA", downconstant: "TGCTAATGCGTACTGACTAG", constants: “all”, constantmismatches: 0.2, barcode_length: 60. Barcode reads were aligned to the SPLINTR GFP reference library.[9] Barcodes supported by most UMI counts per cell were retained. Barcodes from spatial data were aggregated to bin size 20 to approximate single-cell resolution.

Software availability

BARtab and bartools are both freely available at https://github.com/DaneVass/bartools and https://github.com/DaneVass/BARtab under MIT and GPL3 licenses respectively. Extensive documentation is available at https://danevass.github.io/bartools. Bartools can be installed into R v3.5 or above using instructions that can be found at https://github.com/DaneVass/bartools. Bartools integrates with other packages available from the Bioconductor project and utilises functions and object classes from edgeR,[34] ineq[40] and vegan.[39] Graphical functions from base R and ggplot2 are also utilised within BARtab and bartools.

BARtab can be installed into macOS or UNIX environments compatible with the Nextflow workflow manager v23.04 and later.[23] BARtab depends on common bioinformatic tools including Samtools,[27] Bowtie,[25] Starcode,[24] FLASh,[26] FastQC,[65] fastp,[29] UMI-tools,[66] MultiQC,[58] and GNU parallel,[57] and requires Python v3.8 or greater. We provide a Docker image compatible with Singularity to facilitate pipeline portability across systems. All dependencies required to successfully run the pipeline are also available from the conda and bioconda projects.[60] Available parameters for BARtab are specified in the documentation (https://github.com/DaneVass/BARtab/blob/main/README.md) which also details approaches for pipeline and software dependency installation via Singularity,[61] Docker or conda environments.

QUANTIFICATION AND STATISTICAL ANALYSIS

BARtab workflow implementation

BARtab is implemented as Nextflow[23] DSL2 pipeline for parallelization, portability and reproducibility. Various filtering, adapter trimming and alignment/clustering settings are fully parameterised in BARtab promoting user customizability and pipeline flexibility. The pipeline contains two main workflows supporting (i) population-level cellular barcoding datasets or (ii) single-cell expressed cellular barcoding datasets. The pipeline can be run in a reference-based or reference-free mode, either utilizing a reference library of known barcodes or relying on a Levenshtein clustering approach implemented in Starcode[24] to correct for errors from PCR and sequencing.

BARtab population-level workflow

For population-level analyses, either paired-end or single-end fastq files can be provided as input. Paired-end reads with a user-defined minimum overlap length are merged by FLASh.[26] Reads that do not achieve a user-defined Phred score across a user defined percentage of the read or a minimum complexity are filtered out by fastp.[29] Next, reads containing user-defined sequences flanking the barcode (known as “constant regions”) are identified and trimmed by cutadapt[28] leaving only the variable barcode sequence. The parameterization of BARtab offers a lot of flexibility at this stage, allowing users to trim only upstream, only downstream, both up and downstream or upstream with optional downstream constant regions. This maximises pipeline flexibility for different barcode construct designs and sequencing strategies. In addition, the allowed error rate within constant regions and the minimum required coverage of each constant region can be specified by the user. The latter allows for trimming of partial adapters at either end of the sequence which can arise if the sequenced read does not cover the entire constant region or if there is variability in

barcode position within the sequenced read. Given the barcode length and sequencing method (whole or partial coverage of the barcode), the expected barcode length and minimum barcode read length can also be specified by users.

If a reference library containing a list of known barcodes (in fasta format) is supplied, barcode containing reads are aligned using Bowtie.[25] Users can allow up to 3 mismatches (the maximum allowed by Bowtie) at this stage. If barcodes have a fixed length, only alignments to the start or end of a reference sequence are retained (see single-cell workflow). As an optional step, unmapped reads can be clustered using Starcode (as for the reference-free workflow) and output separately. Finally, barcode read count tables per sample are generated by Samtools and custom R scripts. If no reference is provided, reads shorter than the defined minimum allowed read length are removed. Remaining barcode reads are trimmed to match the length of the shortest read unless both up and downstream constant regions are trimmed, in which case the full-length barcode is retained. This allows the use of variable barcode lengths within a single sample. Reads are then clustered and merged within a user-defined Levenshtein distance by Starcode.[24] Finally, barcode read counts from alignment or clustering approaches are compiled across samples, resulting in a final counts table containing all barcodes as rows and all samples as columns.

BARtab single-cell workflow

The single-cell workflow can extract lineage barcodes from (i) amplicon-sequencing data (as paired-end fastq input) from single cell library cDNA template DNA or (ii) directly from pre-processed and aligned single-cell datasets (BAM file format). For extraction of lineage barcodes from single-cell amplicon-seq data, read pairs are firstly filtered for valid cell barcodes using UMI-tools.[66] BARtab can perform cell calling (i.e., identifying droplets likely to contain cells) using the functionality available within UMI-tools. Alternatively, a whitelist of known cell barcodes can be provided (e.g., from scRNA-seq data pre-processed with Cell Ranger by 10X Genomics or similar). Subsequently, input paired reads are filtered for sequence quality (as for the population-level workflow). Reads containing user-defined constant regions flanking the lineage barcode are identified and trimmed (as for the population-level workflow).

Lineage barcode containing reads can either be aligned to a reference library with Bowtie (as for the population-level workflow) or clustered with Starcode-umi.[24] The latter is a wrapper for Starcode to cluster UMI-tagged sequences. BARtab then performs PCR chimera identification and removal. PCR chimeras can arise due to early DNA polymerase termination during PCR extension, and mispriming of the partial amplicon to a different template in subsequent cycles, leading to a hybrid amplicon of a cell barcode and UMI with a different lineage barcode. Here, for each cell barcode-UMI combination, only the lineage barcode supported by most reads is retained and ambiguous ties are removed. Next, UMI error correction is performed by UMI-tools and UMIs for accepted cell barcode-lineage barcode combinations are collapsed within a user-defined Levenshtein distance. Finally, the lineage barcodes detected within individual cells, as well as the number of UMIs corresponding to each lineage barcode are combined into as a table of counts per sample. To address potential noise from ambient RNA in droplets, lineage barcodes can be filtered using a user-defined UMI count threshold. Lineage barcodes with a UMI count less than a user-defined fraction of the most abundant lineage barcode in that cell (by total UMIs) can also be removed.[10] Lineage barcodes and their UMI counts are collapsed as comma separated lists for each cell barcode to provide barcode annotation at the single-cell level. In addition, QC plots of unfiltered and filtered lineage barcode counts are reported.

When extracting barcodes from pre-processed scRNA-seq data, cell barcode extraction and read filtering can be skipped. Instead, BAM files generated by scRNA-seq processing pipelines like Cell Ranger by 10X Genomics or STARsolo[43] (where the cell barcode and UMI are annotated in the read header) can be provided to BARtab. Due to the random fragmentation of reads during scRNA-seq library preparation, trimming can be performed so that reads with only the upstream, only the downstream or both constant regions are identified. This improves the recovery of barcodes from this data. However, for barcodes extracted in this way only the reference-based quantification approach is supported by BARtab. For both population-level and single-cell level workflows, MultiQC[58] is used to compile log files from tools used in the pipeline into a comprehensive pipeline run report that is output alongside sample counts tables.

BARtab performance comparison – Population level data

A dataset consisting of 22 population level barcode sequencing samples from Goyal et al.[30] was downloaded from Figshare (https://figshare.com/articles/dataset/FateMap_Paper_datasets_3_Goyal_et_al_2021_Biorxiv_/22806494). The dataset was reanalysed with the published Rewind/TimeMachine code (https://github.com/arjunrajlaboratory/timemachine, commit 9b8c24f) from the same publication using default settings in raw read mode, or with pycashier version v23.1.2 and BARtab v1.4 using settings to mimic these conditions as closely as possible. TimeMachine additionally required the stagger length per sample which was derived from the raw read sequences.

BARtab was run using default parameters except for the following: up_coverage: 10, down_coverage: 10, min_readlength: 40, constants: "both", cluster_distance: 8, cluster_ratio: 5, upconstant: "GACTAAACGCGCTACTTGAT" and downconstant: "ATCCTACTTGTACAGCTCGT".

pycashier was run using the following parameters: quality = 20, unqualified_percent = 20, error = 0.1, length = 100, upstream_adapter = "GACTAAACGCGCTACTTGAT", downstream_adapter = "ATCCTACTTGTACAGCTCGT", ratio = 5, distance = 8, filter_percent = 0 and offset = 8.

BARtab, pycashier and Rewind/TimeMachine differ in the way they handle extraction of barcodes of variable length. For the comparative analyses, we set length parameters of BARtab and pycashier to match those applied by Rewind/TimeMachine as

closely as possible, within the constraints of each software. The Rewind/TimeMachine method by default extracts barcodes of variable length, and length restrictions are defined by the identification and trimming of the GFP primer site and downstream constant region within the FateMap construct.[13,30] Based on published FateMap barcode design features: sequenced read length of 150bp, a 0-6bp stagger, 6bp UMI, 22bp GFP primer region, and 15bp of the downstream constant region in bases 80–150; we deduced that FateMap barcodes can exist in a length range of 40-107bp. In contrast, pycashier[21] allows for extraction of variable length barcodes but restricts the accepted range of barcode lengths to be +/− the Levenshtein distance used for clustering barcode reads. Since the Levenshtein distance used by the Rewind/TimeMachine method is 8, this is equivalent to an allowed barcode length range of 92-108bp in pycashier. BARtab takes a more flexible approach by applying user-defined minimum and maximum barcode length parameters and filters reads that fall outside this defined size range. To match the size restriction of TimeMachine, BARtab was run with a minimum barcode length of 40bp. The maximum barcode length was set to 130 which corresponds to the presence of at least 10bp of the GFP primer and downstream constant region required by Rewind/TimeMachine.

For the runtime evaluation, we ran TimeMachine with default settings which clusters barcodes using the barcode read counts. To ensure we ran TimeMachine correctly, we also clustered barcodes on the UMI counts and observed a 100% overlap of identified barcodes with published results. For the comparison of detected barcodes between TimeMachine, BARtab and pycashier, we first aggregated 4 replicates per sample by averaging barcode read counts for BARtab and pycashier. We then filtered barcodes present with at least 0.001% within any sample for all three methods. For the analysis of concordance of barcode quantification between BARtab and TimeMachine, we calculated the spearman and pearson correlation for each sample, considering all barcodes detected in the respective sample by any of the two methods.

BARtab performance comparison – Single-cell data

Fastq files from Goyal et al. 2023[30] containing single-cell amplicon sequencing data from dataset "FM0-2" were downloaded from Figshare https://figshare.com/articles/dataset/FateMap_Paper_datasets_2_Goyal_et_al_2021_Biorxiv_/22802888?file=40535864. Lists of corresponding cell barcodes were downloaded from GEO GSM7434409, GSM7434410, GSM7434411, GSM7434412. We observed that provided processed data, available on Figshare https://figshare.com/articles/dataset/FateMap_Paper_datasets_1_Goyal_et_al_2021_Biorxiv_/22798952?file=40534928, contained cell barcodes not present in the whitelists. Therefore, we re-ran the reported FateMap analysis pipeline on the FM0-2 input data using the code provided with the manuscript https://github.com/arjunrajlaboratory/FateMap_Goyal2023/tree/main/extractionScripts/10XScripts/10XBarcodeMatching/10XBarcodeMatching-master. BARtab was run on the FM0-2 dataset using one pair of fastq files (fastq files from individual lanes were concatenated) and one cell barcode whitelist per sample as input. To tailor BARtab to the FateMap data and match the FateMap configuration as close as possible, we chose following BARtab runtime parameters: mode: "single-cell", input_type: "fastq", complexity_threshold: 65, minqual: 15, pctqual: 70, constants: "up", upconstant: "GGACGAGCTGTACAAGTAGG", up_coverage: 20, min_readlength: 50, constantmismatches: 0.2, cluster_distance: 8, cluster_ratio: 5, cb_umi_pattern: "CCCCCCCCCCCCCCCCNNNNNNNNNN". Default values were used for all other parameters.

As per the reported FateMap analysis pipeline, we retained read pairs containing whitelisted 16bp cell barcodes and a 10bp UMI in read 1. FateMap removes reads that contain 6 or more bases with a Phred score of less than 15 in the stagger and GFP sequence. To match this as closely as possible we set BARtab to remove reads with a Phred score of less than 15 in more than 30% of the read. FateMap also removes reads containing strings of 4 consecutive bases. Given the semi-random repeating "WSN" pattern of the FateMap barcode, we applied a complexity threshold of 65%, defined as minimum percentage of bases that are different from their next base (base[i] ! = base[i+1]). As per FateMap, BARtab was set to retain reads containing the full 20bp upstream constant GFP region, allowing 4 mismatches (20%). Reads containing at least 50 of the 100 bases of the Rewind/FateMap barcode following the constant region were retained. As per FateMap, BARtab was set to trim barcode reads to 50bp length before clustering.

FateMap identifies unique cell barcode-UMI-lineage barcode combinations and clusters those lineage barcodes across all samples within an experiment. Sphere clustering is applied within an Levenshtein distance of 8. In contrast, BARtab clusters barcode sequences for each sample using all reads that pass quality control thresholds, including PCR duplicates. In Starcode, which is used by BARtab for barcode read clustering, a message passing clustering algorithm is used with a Levenshtein distance of 8 and cluster ratio of 5. After identification and removal of PCR chimeras, BARtab collapses UMI per barcode per cell within an Levenshtein distance of 1. BARtab results were loaded into R using the bartools function readBartabCounts(). To evaluate the cell annotation performance of BARtab in comparison to FateMap, we applied various UMI count thresholds using the bartools function filterBarcodes() and compared the percent of cells with a single barcode annotated. To compare the clone sizes between FateMap and BARtab methods, we applied a minimum UMI threshold of 15 (FateMap) and 5 (BARtab) and only retained cells annotated with a single barcode. The Pearson correlation of clone sizes was then calculated for each of the 4 samples in the FM0-2 dataset.

Cell Reports Methods, Volume *4*

Supplemental information

Analysis of synthetic cellular barcodes in the genome and transcriptome with BARtab and bartools

Henrietta Holze, Laure Talarmain, Katie A. Fennell, Enid Y. Lam, Mark A. Dawson, and Dane Vassiliadis

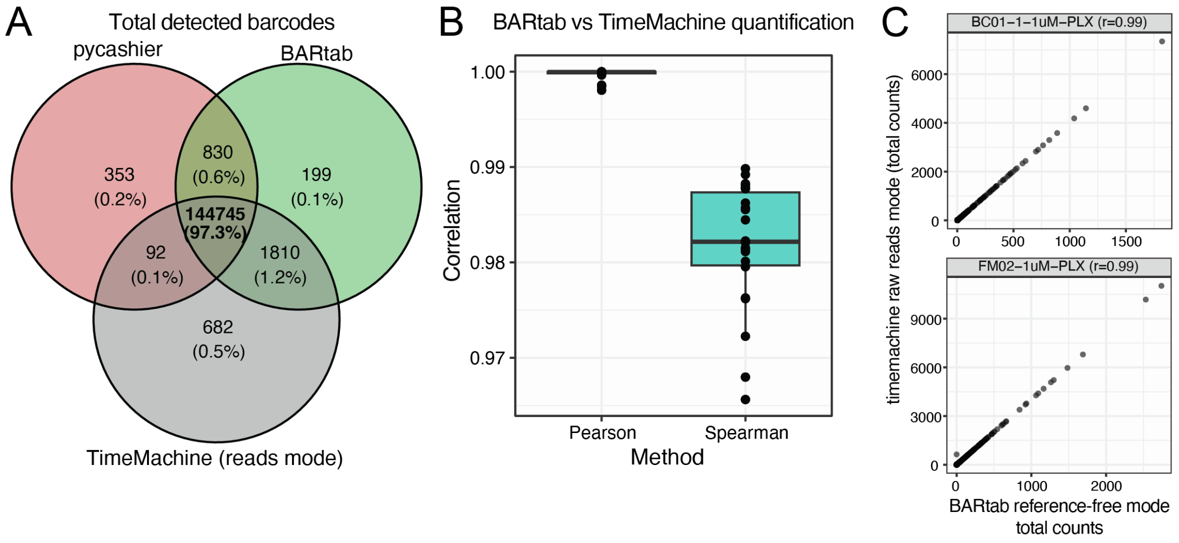

Figure S1. BARtab performance comparison – population-level data. Related to Figure 2. A) Venn diagram of total barcodes detected by BARtab, pycashier and TimeMachine (reads mode) across the 22 samples of the Goyal et al. dataset after filtering barcodes below 0.001% within a sample. **B)** Pearson and Spearman correlation of BARtab barcode quantification compared to TimeMachine (reads mode). Boxplots indicate mean and interquartile range with whiskers extending 1.5x the IQR. All samples are shown as individual points. **C)** Exemplar scatter plots for two samples from the Goyal et al. dataset showing total counts per barcode from BARtab and TimeMachine, Spearman's rank correlation coefficient in parentheses (reads mode).

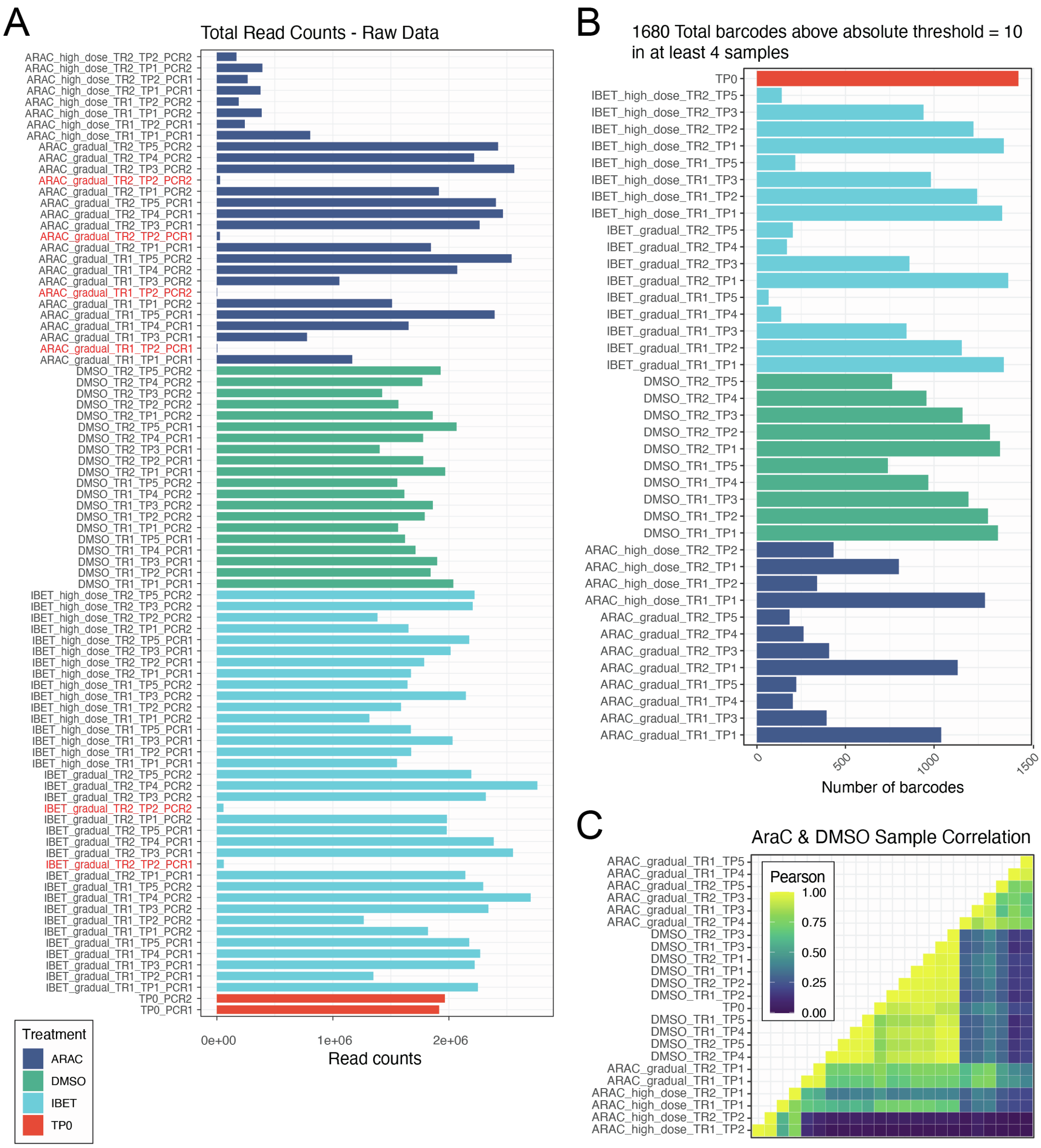

Figure S2. Additional quality control metrics for the dose escalation dataset. Related to Figure 2. A) Total number of read counts per sample for the dose escalation dataset. Poor quality samples identified using 5th percentile-based outlier detection are highlighted in red. **B)** Total number of lineage barcodes detected per sample post quality control and filtering steps for the dose escalation dataset. **C)** Pairwise Pearson correlation heatmap of biological replicate samples (indicated by TR1/2) for AraC and DMSO treatment groups in the dose escalation dataset. TR = technical replicate. TP = time point. TP0 = time point 0 / baseline sample.

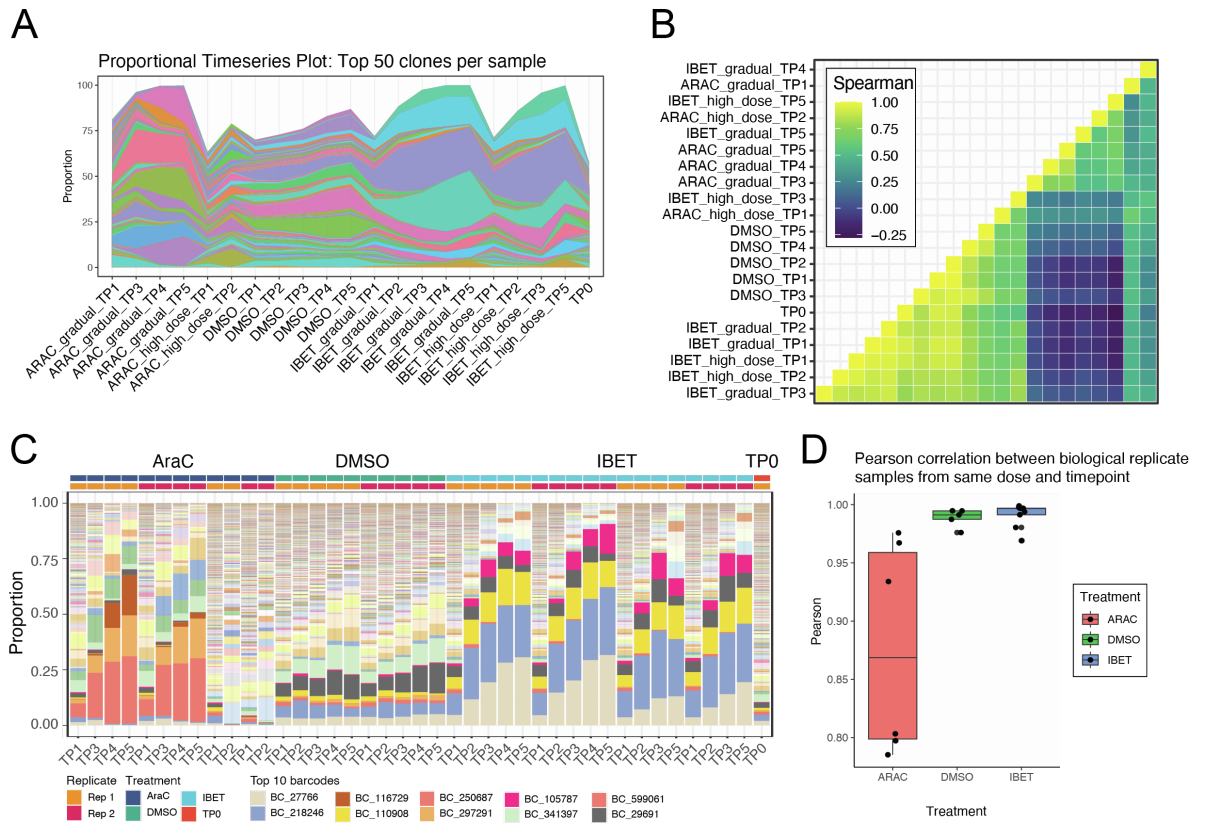

Figure S3. Additional global barcode abundance analyses for the dose escalation dataset. Related to Figure 3. A) Timeseries plot of the most abundant 50 barcodes across all samples within biological replicate 1 from the dose escalation dataset. **B)** Sample-sample pairwise Spearman correlation matrix of the dose escalation dataset, hierarchically clustered according to sample similarity. **C)** Stacked histogram of proportional barcode abundance for each sample in the dose escalation dataset. Treatment and replicate groups are indicated by the rows above the plot, the timepoint is indicated on the x-axis. The top 10 most abundant clones across the entire dataset are indicated. **D)** Boxplot of Pearson correlations between matched samples from replicate 1 and replicate 2 of the dose-escalation dataset for each of the three treatment groups. Boxplots show the mean and inter-quartile range (IQR). Whiskers extend 1.5x the IQR. Points indicate Pearson correlation values for individual pairs of samples. TP = time point. TP0 = time point 0 / baseline sample.

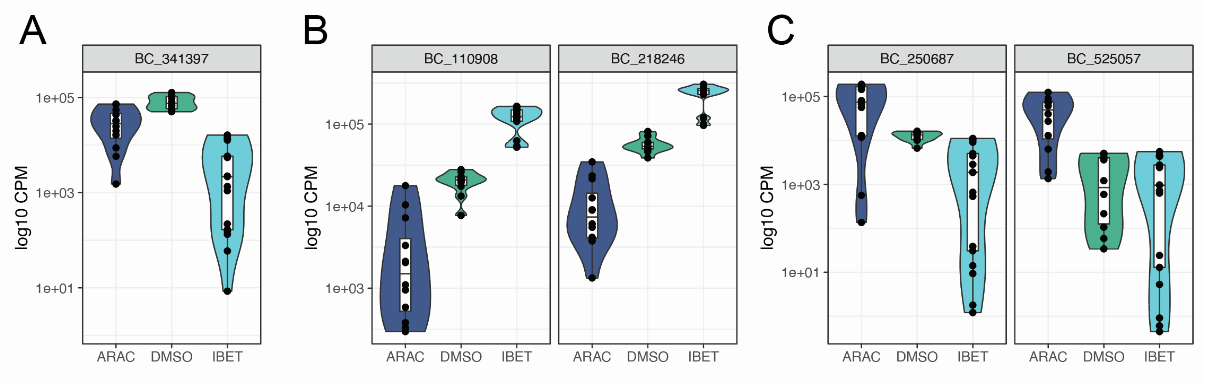

Figure S4. Individual barcode-level analysis. Related to Figure 3. Violin plots of log10 transformed counts per million (CPM) for selected barcodes from the dose escalation dataset predominant in the **(A)** vehicle (DMSO) condition, **(B)** IBET treatment condition and, **(C)** AraC treatment condition. Inset boxplots show the mean and inter-quartile range (IQR). Whiskers extend 1.5x the IQR. Points indicate CPM values for individual samples.

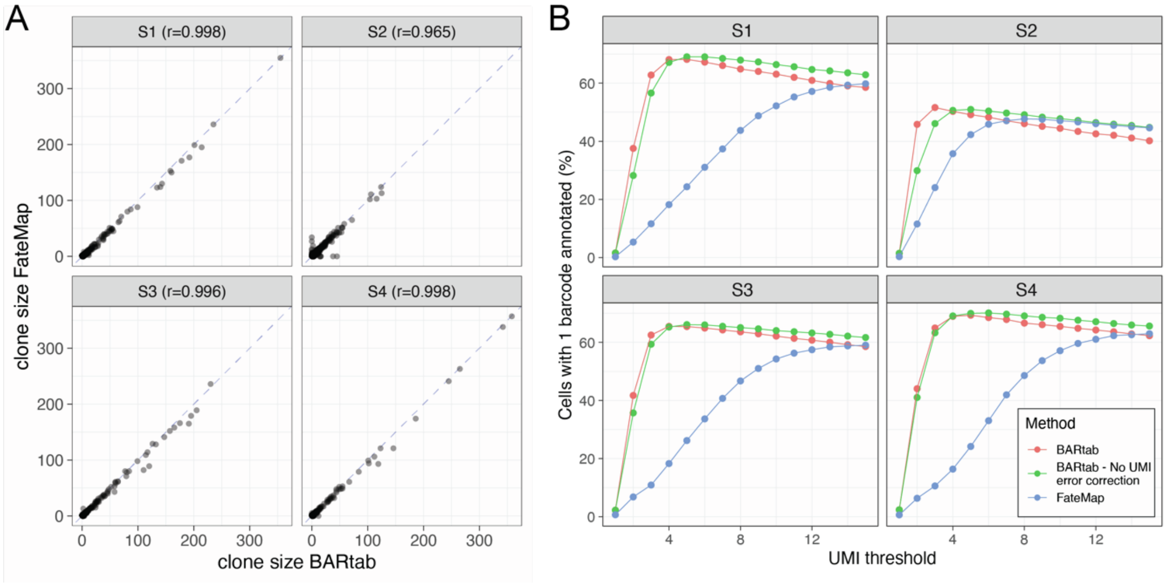

Figure S5. BARtab performance comparison – single cell data. Related to Figure 4. A) Comparison of clone sizes from single cell barcode annotation results by FateMap vs. BARtab. Samples 1-4 from the Goyal et al. 2023 FM02 dataset are shown. BARtab results are filtered for UMI threshold = 5. FateMap was run according to published parameters (UMI threshold = 15). Pearson correlation coefficient in parentheses, dashed line is x=y. **B)** Total number of cells annotated with single lineage barcodes by FateMap or BARtab across a range of UMI thresholds for each of the four samples in the Goyal et al. 2023 FM02 dataset. FateMap was run according to published parameters. BARtab was run with (red line) or without (green line) UMI sequence error correction.

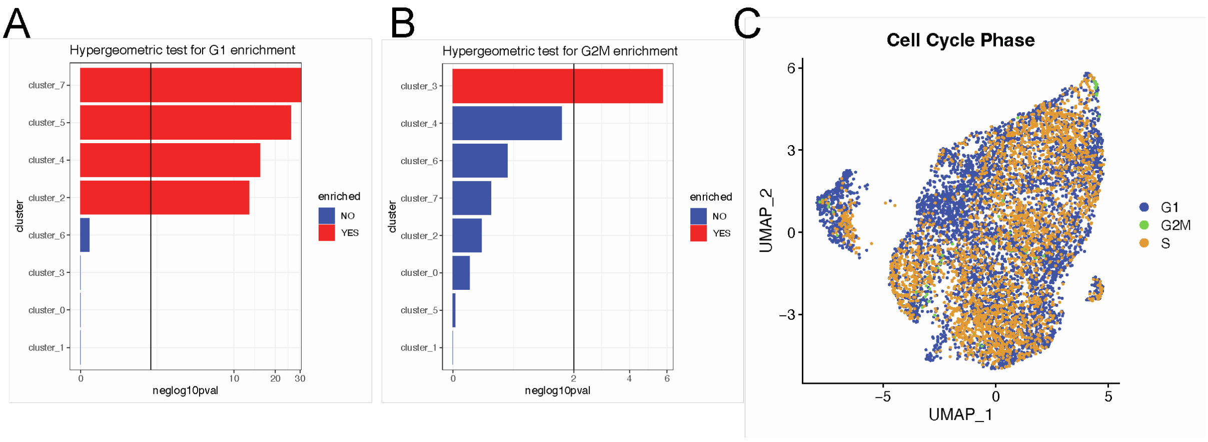

Figure S6. The plotClusterEnrichment function in *bartools* is agnostic to the grouping variable. Related to Figure 4. Hypergeometric testing for enrichment of cell cycle label G1 **(A)** and G2M **(B)** for cells within each Louvain cluster in the single-cell dataset. **C)** UMAP visualisation of the single-cell dataset with cells within each cell cycle phase highlighted.

Table S1: Example population-level cellular barcoding experiment sample sheet. Related to Figure 2.

sample	technical_rep	treatment	dose	timepoint	files
Treatment_rep1-1	1	Drug	1000nM	week_1	path/to/counts/file.txt
Treatment_rep1-2	2	Drug	1000nM	week_1	path/to/counts/file.txt
Treatment_rep2-1	1	Drug	1000nM	week_4	path/to/counts/file.txt
Treatment_rep2-2	2	Drug	1000nM	week_4	path/to/counts/file.txt
Vehicle_rep1-1	1	Vehicle	Vehicle	week_1	path/to/counts/file.txt
Vehicle_rep1-2	2	Vehicle	Vehicle	week_1	path/to/counts/file.txt
Vehicle_rep2-1	1	Vehicle	Vehicle	week_4	path/to/counts/file.txt
Vehicle_rep2-2	2	Vehicle	Vehicle	week_4	path/to/counts/file.txt
Baseline-1	1	Baseline	Baseline	Baseline	path/to/counts/file.txt
Baseline-2	2	Baseline	Baseline	Baseline	path/to/counts/file.txt
